# Evaluation of exploratory fluid biomarkers from a phase 1 senolytic trial in mild Alzheimer’s disease

**DOI:** 10.1016/j.neurot.2025.e00591

**Published:** 2025-04-23

**Authors:** Valentina R. Garbarino, Juan Pablo Palavicini, Justin Melendez, Nicolas R. Barthelemy, Yingxin He, Tiffany F. Kautz, Marisa Lopez-Cruzan, Julia J. Mathews, Peng Xu, Bin Zhang, Afaf Saliba, Nagarjunachary Ragi, Kumar Sharma, Dallin Mason, Samuel Johnson, Suzanne Hendrix, Suzanne Craft, Ronald C. Petersen, Jair Machado Espindola-Netto, Ailing Xue, Tamara Tchkonia, James L. Kirkland, Arash Salardini, Nicolas Musi, Randall J. Bateman, Mitzi M. Gonzales, Miranda E. Orr

**Affiliations:** a Glenn Biggs Institute for Alzheimer’s & Neurodegenerative Diseases, University of Texas Health Science Center at San Antonio, San Antonio, TX, USA; b Department of Cell Systems and Anatomy, University of Texas Health Science Center at San Antonio, San Antonio, TX, USA; c Department of Medicine, University of Texas Health Science Center at San Antonio, San Antonio, TX, USA; d Barshop Institute for Longevity and Aging Studies, University of Texas Health San Antonio, San Antonio, TX, USA; e Department of Neurology, Washington University School of Medicine, St. Louis, MO, USA; f Tracy Family SILQ Center for Neurodegenerative Biology, St. Louis, MO, USA; g Department of Psychiatry, University of Texas Health Science Center at San Antonio, San Antonio, TX, USA; h Department of Genetics and Genomic Sciences, Icahn School of Medicine at Mount Sinai, New York, NY, USA; i Mount Sinai Center for Transformative Disease Modeling, Icahn School of Medicine at Mount Sinai, New York, NY, USA; j Center for Precision Medicine, University of Texas Health Science Center at San Antonio, San Antonio, TX, USA; k Pentara Corporation, Salt Lake City, UT, USA; l Department of Internal Medicine Section on Gerontology and Geriatric Medicine, Wake Forest School of Medicine, Winston-Salem, NC, USA; m Department of Neurology, Mayo Clinic, Rochester, MN, USA; n Department of Physiology and Biomedical Engineering, Mayo Clinic, Rochester, MN, USA; o Department of Internal Medicine, Mayo Clinic, Rochester, MN, USA; p Department of Neurology, University of Texas Health Science Center at San Antonio, San Antonio, TX, USA; q Department of Medicine, Cedars-Sinai Medical Center, Los Angeles, CA, USA; r Department of Neurology, Cedars Sinai Medical Center, Los Angeles, CA, USA; s St Louis VA Medical Center, St Louis, MO, USA

**Keywords:** Alzheimer’s disease, Senolytics, Clinical trial, Biofluids, Biomarkers

## Abstract

Senescent cell accumulation contributes to the progression of age-related disorders including Alzheimer’s disease (AD). Clinical trials focused on cellular senescence are in early stages and have yet to establish reliable outcome measures reflecting senescent cell burden or response to senolytics, therapeutics that clear senescent cells. Results from the first open-label trial of senolytics, dasatinib plus quercetin (D ​+ ​Q), in older adults (N ​= ​5) with early AD demonstrated central nervous system penetration of dasatinib and favorable safety and tolerability. Herein, we present exploratory analyses of senescence and AD-associated analytes in blood, cerebrospinal fluid (CSF) and urine from this study in effort to guide biomarker development for future senolytic trials. Immunoassays, mass spectrometry and transcriptomics were performed and changes in analyte levels were assessed from baseline to post-treatment using paired t-tests. Targeted cytokine and chemokine analyses revealed increases in plasma fractalkine and MMP-7 and CSF IL-6 from baseline to post-treatment. Mass spectrometry indicated stable levels of amyloid β and tau proteins in CSF, unchanged urinary metabolites, and modest treatment-associated lipid profile changes. Targeted transcriptomic analysis of peripheral blood mononuclear cells indicated downregulation of inflammatory genes including *FOS, FOSB, IL1β, IL8, JUN, JUNB, PTGS2.* The levels and treatment responses of the analytes identified here may help inform trial design and outcomes for senolytic studies. Independent validation will be necessary to develop standardized biomarker panels across senolytic trials for AD.

ClinicalTrials.gov: NCT04063124.

## Introduction

Cellular senescence refers to a complex stress response that culminates in a change in cell state [1]. Cells enter a fate of senescence when irreparable cellular damage fails to induce apoptosis and instead triggers the upregulation of both apoptotic and survival pathways, with the balance shifting toward survival. Once formed, senescent cells permanently remain in tissues unless cleared by the immune system. The accumulation of these dysfunctional cells and their accompanying pro-inflammatory senescence associated secretory phenotype (SASP) contribute to many age-associated pathogenic processes and diseases, including amyloid β (Aβ) and tau pathologies of Alzheimer’s disease and related dementias (AD; ADRDs) [[2], [3], [4], [5]].

Targeted removal of senescent cells has been achieved by a combination therapy consisting of dasatinib (D), a broad-spectrum tyrosine kinase inhibitor, plus quercetin (Q), a flavonoid (D ​+ ​Q) [6]. This therapeutic approach, referred to as senolytic, improves healthy lifespan in various laboratory model systems, including ADRD transgenic mice [2,4,7]. D ​+ ​Q has demonstrated successful reduction in senescent cell burden, inflammatory SASP, AD-related neuropathological burden of tau-containing neurofibrillary tangles (NFTs) [2] and Aβ plaques [4], and age-associated cognitive deficits [8,9]. The promising results have motivated clinical testing of senolytics in human studies for AD and other age-associated diseases (for review: [[10], [11], [12], [13]]; ongoing AD trials: SToMP-AD: NCT04685590 and ALSENLITE: NCT04785300).

Open label phase 1 human trials using D ​+ ​Q were first tested in the context of idiopathic pulmonary fibrosis (IPF) [14] and diabetic kidney disease [15]. Both studies reported that D ​+ ​Q was safe and well tolerated. We recently completed a 12-week, phase 1 open-label pilot study, Senolytic Therapy to Modulate the Progression of AD: SToMP-AD; NCT04063124. Results indicated direct brain exposure to D and favorable safety and tolerability in participants with early-stage symptomatic AD [16]. To advance these phase 1 safety trials, and evaluate target engagement and efficacy in phase 2, methodologies are needed to monitor the presence, abundance, and clearance of senescent cells.

Major advances have been made in fluid and imaging biomarkers to diagnose and track AD progression in clinical trials [17]; however, biomarker discovery in the senescence field is in its infancy [18]. The heterogeneous cell-type and context-specific phenotype of senescent cells has presented challenges for identifying a universal biomarker [18,19]. Identification of senescent brain cells in preclinical studies has relied on a panel of histological stains and gene expression analyses on postmortem brain tissue. However, target engagement assays for human clinical trials will require non-invasive strategies using accessible biological samples. Therefore, we biobanked blood, CSF and urine from the phase 1 trial at baseline and after treatment for exploratory outcome analyses.

Prior open label pilots reported a D ​+ ​Q-associated reduction in circulating SASP factors [14], senescent cells in adipose and skin biopsies [15] and modest change in SASP-associated IL-6 [16]. To harmonize protocols and outcomes across senolytic studies, we used the same laboratory and methods to analyze SASP factors as the prior D ​+ ​Q human trials [14,15]. Additionally, we included quantitative assessment of cytokines and chemokines linked to SASP measured in plasma, CSF, and urine; mass spectrometry analysis of AD-associated Aβ and phosphorylated tau isoforms in CSF; metabolite analysis in urine; lipidomic changes in plasma and CSF associated with perturbed lipid metabolism linked to AD [20]; and gene expression changes in peripheral blood mononuclear cells (PBMCs) using a custom transcriptomics panel relevant to chronic stress termed the conserved transcriptional response to adversity (CTRA) [21].

The survey of analytes presented here is both an attempt to begin standardizing outcomes by measuring the SASP factors using the same laboratory and methods as earlier studies, and broaden the analyses in search of additional biomarkers across biofluids that respond to senolytics in an AD relevant population. While the trial was not powered to determine disease modification through biomarker changes, the levels and variance of these analytes may be used to aid the design of biomarker panels, outcome measures, and sample size determinations of future phase 2, placebo-controlled efficacy trials.

## Methods

### Study design

The full study protocol [22], as well as the detailed results from the initial reporting of results of the SToMP-AD trial, have been separately published [16]. In brief summary, five individuals with early-stage AD were recruited to participate in an open-label trial which provided dasatinib (100 ​mg, Sprycel, Bristol Meyers Squibb) and quercetin (1000 ​mg, Thorne Research) orally on an intermittent dosing schedule for three months. The trial was conducted in compliance with all relevant ethical regulations and the Guideline for Good Clinical Practice. D ​+ ​Q were administered under Investigation New Drug (IND) 143945-0006 (to N.M.). The study protocol was approved by the UT Health San Antonio Institutional Review Board (IRB). All participants provided written informed consent with an appropriate legally authorized representative.

### Biospecimen collection and storage

All plasma, CSF, and urine biospecimens utilized for these analyses were collected under fasting conditions as described previously, at baseline (Visit 1) and post-treatment (Visit 9), the morning of the second day of the final drug administration cycle [16]. Peripheral blood mononuclear cell (PBMC) isolation was performed from blood collected in BD Vacutainer CPT Mononuclear Cell Preparation (CPT) Sodium Heparin tubes (Franklin Lakes, NJ) according to the manufacturer’s protocol. The resulting PBMCs were stored in three, 1 ​ml aliquots containing heat inactivated fetal bovine serum (Corning, NY) with 10 ​% dimethyl sulfoxide (Corning, NY). The PBMCs were stored overnight at −80 ​°C in a Mr. Frosty container (Nalgene, Rochester, NY) before final storage in a liquid nitrogen freezer.

### Mass spectrometry for amyloid and tau cerebrospinal fluid biomarkers

Previously published methods were utilized to measure CSF Aβ [23], tau and ptau peptides, residues [24], and HJ32.11-MTBR-tau microtubule binding regions [25]. A total of 27 Aβ and tau relevant markers were assessed by this method, without correction for multiple comparisons corrections due to the small sample size.

### Inferring blood-brain-barrier integrity with drug and biomarker correlation

Levels of CSF NfL and D penetrance into the CSF were assayed as described in Gonzales et al., 2023 [16]. A simple linear regression between baseline NfL levels and post-treatment D levels in the CSF were assessed for each participant.

### Senescence associated secretory factors in plasma, cerebrospinal fluid, and urine

Baseline versus post-treatment levels of plasma, CSF, and urine biomarkers associated with SASP were evaluated at the Facility for Geroscience Analysis (FGA) at Mayo Clinic. This laboratory is part of the NIH-funded Translational Geroscience Network. Duplicate samples were analyzed using either the FLEXMAP3D Machine (Luminex) or the Ella Automated Immunoassays (Protein Simple, Bio-Techne) platforms with commercially available immunoassay kits (R&D Systems, Bio-Techne). Based on the abundance of the targeted factors, bead region, and antibody compatibility, the targets were organized into 18, 10, 6, and 5 plex plates for plasma and 15, 13, and 5 plex plates for urine. Proteins with very low abundance were measured using the ELLA Automated Immunoassay (Protein Simple/Bio-Techne) with cartridges purchased from Protein Simple/Bio-Techne. All assays were conducted according to the manufacturer’s instructions. Adiponectin was excluded from the 15-Plex urine panel due to compatibility issues with the assay beads. Urine protein levels were normalized to creatinine levels for each participant at each timepoint using commercially available kits (R&D Systems/Bio-Techne). Overall, there were 27 plasma proteins, 8 CSF proteins, and 32 urinary proteins included in the analysis. The baseline and post-treatment levels were compared using paired-sample *t*-tests to assess the effect of the senolytic treatment, without correction for multiple comparisons due to small sample size. One participant was unable to provide a baseline urine sample, so the associated post-treatment time point was excluded from the analyses. Values below the assays' detection limit, resulting in the absence of a matching paired sample, were excluded from the paired *t*-test analyses.

### Metabolite analysis in urine samples

A panel of 17 urinary metabolites were measured with urine samples collected at baseline and post-treatment from 4 of the 5 study participants. Mass spectrometry (MS) protocols were slightly modified from previous publications [26,27]. Briefly, for LC/MS/MS we utilized a Thermo Q Exactive HF-X Orbitrap mass spectrometer with a Thermo Vanquish HPLC system, auto-injecting a 5 ​μL urine sample. For chromatography, we used an Agilent ZORBAX HILIC PLUS column with a mobile phase of components A (10 ​mM ammonium bicarbonate, 0.05% formic acid in Millipore water, pH ​= ​4.2) and B (0.1% formic acid in acetonitrile), with flow rate flow rate of 0.3 ​mL/min. The gradient ran for 12 ​min. MS settings included a 4300 ​V spray voltage, nitrogen gas, ion transfer tubes, and auxiliary heater at 320°C and 30°C, respectively. PRM mode was positive polarity. Data were processed using Xcalibur Quant Browser, comparing peak areas to internal standards (A/IS ratio) and a standard curve (0.01–100 ​μM) for concentration determination.

### Multidimensional mass spectrometry-based shotgun lipidomics in plasma and cerebrospinal fluid

Total protein concentrations for plasma and CSF samples were determined using bicinchoninic acid (BCA) protein assay (Thermo Fisher Scientific). Lipids were extracted by a modified procedure of Bligh and Dyer extraction in the presence of internal standards, which were added based on plasma or CSF volume for each sample as previously described [28]. Lipid analyses was expressed and analyzed as per total protein content and sample volume. Lipids were assessed using a triple-quadrupole mass spectrometer (Thermo Scientific TSQ Altis) and a Quadrupole-Orbitrap™ mass spectrometer (Thermo Q Exactive™) equipped with a Nanomate device (Advion Bioscience Ltd., NY, USA) as previously described [29,30]. Briefly, diluted lipid extracts were directly infused into the electrospray ionization source through a Nanomate device, signals were averaged over a 1-min period in the profile mode for each full scan MS spectrum. For tandem MS, collision gas pressure was set at 1.0 mTorr, but the collision energy varied with the classes of lipids. Similarly, a 2–5 ​min period of signal averaging in the profile mode was employed for each tandem MS mass spectrum. All full and tandem MS mass spectra were automatically acquired using a customized sequence subroutine operated through Xcalibur software. Data processing including ion peak selection, baseline correction, data transfer, peak intensity comparison, ^13^C deisotoping, and quantitation were conducted using a custom programmed Microsoft Excel macro after considering the principles of lipidomics [31]. A total of 194 lipid species were measured in plasma and 79 lipid species were measured in CSF. Given our small sample size and lack of statistical power to resolve putative sex-specific treatment effects, paired analysis of all subjects (males ​+ ​females) was performed to focus on the effects of senolytic treatment in relation to the participant’s baseline.

### RNA preparation for transcriptomic analyses

To analyze participant PBMC samples for senolytic induced changes in genes included in the CTRA transcriptomic profile, RNA was isolated from frozen PBMC samples using the QIAGEN protocol for isolation of total RNA from PBMCs outlined with the RNeasy Mini Kit (ca. no. 74104). RNA 260/280, 260/230, and RNA concentration were assessed using NanoDrop, and samples were diluted to 10 ​ng/μL with RNAse free water. Samples were prepared following the hybridization protocol assay (NanoString nCounter XT). CTRA genes were assessed in RNA isolated from PBMC specimens using the NanoString nCounter XT CodeSet Gene Expression Panel, custom designed to specifically measure 53 genes which represent the CTRA profile. CTRA gene expression levels were normalized to the following housekeeping genes: *HPRT1*, *PGK1*, *POLR2A*, and *TBP*, while *MAPT* was included as negative control for differential gene expression analysis. Of the 53 a priori selected genes, 45 reached detectable levels by this method, and were evaluated with moderated *t*-test by Robust Empirical Bayes (REBayes) for paired samples.

### Statistical analysis

Baseline to post-treatment changes in plasma and CSF biomarkers were assessed using multiple paired sample *t*-tests in GraphPad Prism version 9.4.1. Paired *t*-tests were two-tailed and significance was determined by *P* ​< ​0.05. Lipidomics statistical analysis was performed using the MetaboAnalyst metadata table and paired one factor modules (https://www.metaboanalyst.ca/). Briefly, lipidomics datasets were transformed (cube root for plasma data expressed relative to protein content and Log_10_ for all other data sets) and scaled (mean centered) so that the data followed a normal distribution. Subclass analyses were performed in GraphPad Prism using multiple paired *t*-tests. Normalized transcriptomic data were analyzed by moderated *t*-test by Robust Empirical Bayes (REBayes) for paired samples in the limma package [32]. Each paired group was treated as a covariate in the design matrix for the paired differentially expressed genes (DEG) analysis between baseline and post-treatment samples. As with the data contained in the original report [16], *P* values were not corrected for multiple comparisons due to the small sample size and exploratory nature of these reported outcomes [33,34]. We applied the ROUT method for identifying outliers to the data. No outliers were identified in any of the significant data or the data displayed as figures. Correlation to explore the relationship between D and NfL was assessed by simple linear regression. Correlation between baseline vs. post-treatment values were assessed by Pearson Correlation if the data were normally distributed according to Shapiro-Wilk test, or Spearman Correlation if non-parametric. Global Statistical Tests (GST) computation analyses were performed using *T*-statistics on three panels of plasma SASP factors generated based off of the protein analytes which were deemed trending or significantly different when comparing baseline to post-treatment D ​+ ​Q treatment in a senolytic trial for diabetic kidney disease (10-analyte panel [35]), SASP factor data achieved by Mesoscale Discovery U-Plex in the previously published results from our trial (13-analyte panel, Extended Data Table 3 in Ref. [16]), and IL-6 alone [16], as a single factor represented in both datasets. A correlation of 0.5 was assumed between the variables within each GST, assuming a moderate relationship between variables. The GST values were used as the treatment effect sizes, and sample size and power analysis calculations were performed with power set to 80 ​% and one-sided alpha level set at 0.025 across four attenuation factors (0.0, 0.2, 0.4, and 0.6) to simulate and predict potential reduction of effect in a real-world population.

## Results

### Study participant sample availability

Five individuals, aged 70–82 years old, with a clinical diagnosis of early-stage dementia due to AD were enrolled in the SToMP-AD pilot study [[16], [22]]. Baseline and post-treatment samples were available from all five participants for tau phosphorylation and microtubule binding domain measures (in CSF), and lipidomics measures (plasma and CSF), while four of the five participant samples were available for Aβ isoform analysis, urine metabolites, and CTRA transcriptomic analysis. Color coding of samples matches those that are described in Table 2 of the parent publication [16], which provides additional information on participant characteristics.

### Senescence associated secretory factors in plasma, CSF, and urine

**Fig. 1:**
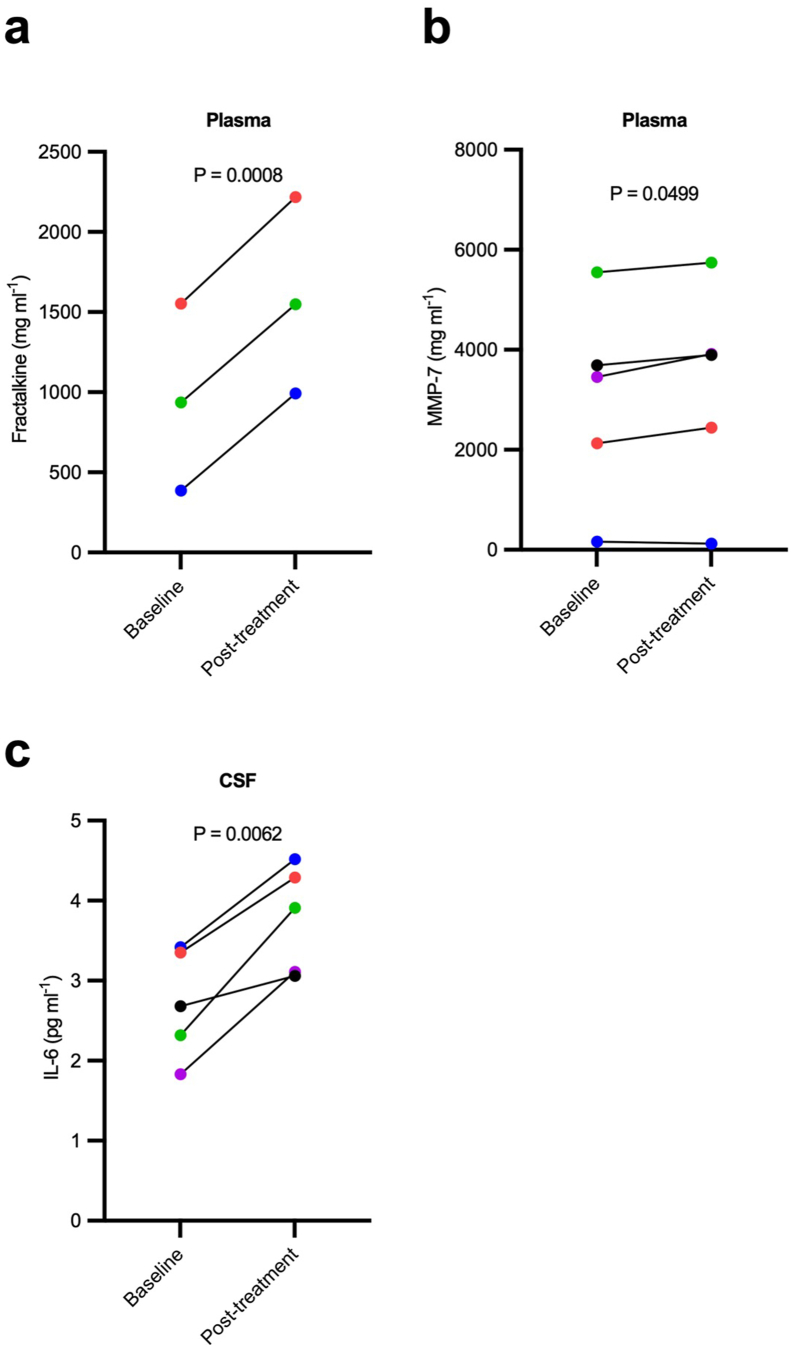
**Inflammatory protein levels altered by dasatinib plus quercetin (D ​+ ​Q) treatment measured by Luminex® protein platform.** a–d, Effect of dasatinib plus quercetin (D ​+ ​Q) on plasma and cerebrospinal fluid (CSF) inflammatory markers. Mean difference (95 ​% CI): a, plasma fractalkine, 629 ​mg/ml (549.90–705.60); b, plasma MMP-7, 226 ​mg/ml (0.198–452.90); c, CSF IL-6, 1.06 ​pg ​ml^−1^ (0.500–1.616). Baseline to post-treatment changes were assessed using two-sided paired sample *t*-tests, *P* ​< ​0.05, N ​= ​3–5, color coded by participant. Mean difference ​= ​post-treatment - baseline; 95 ​% CI, for the post *versus* baseline mean difference. No correction for multiple comparisons was made due to small sample size.

Immunoassays using commercially-available platforms and kits were used to assess multiple SASP factors in plasma, CSF and urine as described in Methods. Baseline to post-treatment paired samples t-tests were performed. Descriptive statistics for each analyte are presented in Supplementary Table 1. There were post-treatment increases in plasma fractalkine (1.65-fold; CV%: 0.0–52.0) and MMP-7 (1.08-fold; CV%: 0.0–63.5), and CSF IL-6 (1.39-fold; CV%: 0.8–7.7) that were statistically significant by paired *t*-test; however, none of the results survived multiple comparisons correction (Fig. 1; Supplementary Table 1). Other analytes that displayed trends toward change at *P ​<* ​0.10 were plasma eotaxin and VEGF (Supplementary Table 1). All analytes measured in urine samples were stable between baseline and post-treatment measures.

Using the previously published plasma SASP factor analyses data available through our study [16] and through the previous open-label senolytic trial in a diabetic kidney disease population [35], we conducted power analysis calculations to determine the sample sizes necessary to achieve 80% power with a one-sided alpha level set to 0.025 in three protein analyte panels (Supplementary Table 2) that may be used to generate a SASP composite score in subsequent trials as was previously done [35]. Our 13-analyte panel calculations indicate that future senolyic trials could expect to achieve an effect size of 0.815 with an assumed attenuation of 0.6 between baseline and post-treatment SASP factor composite scores using as few as 25 participants/arm in an AD relevant trial population compared to 181 participants/arm if only one SASP factor (e.g. IL-6) were considered (Supplementary Table 2).

### Aβ and tau biomarker measures in CSF

**Fig. 2:**
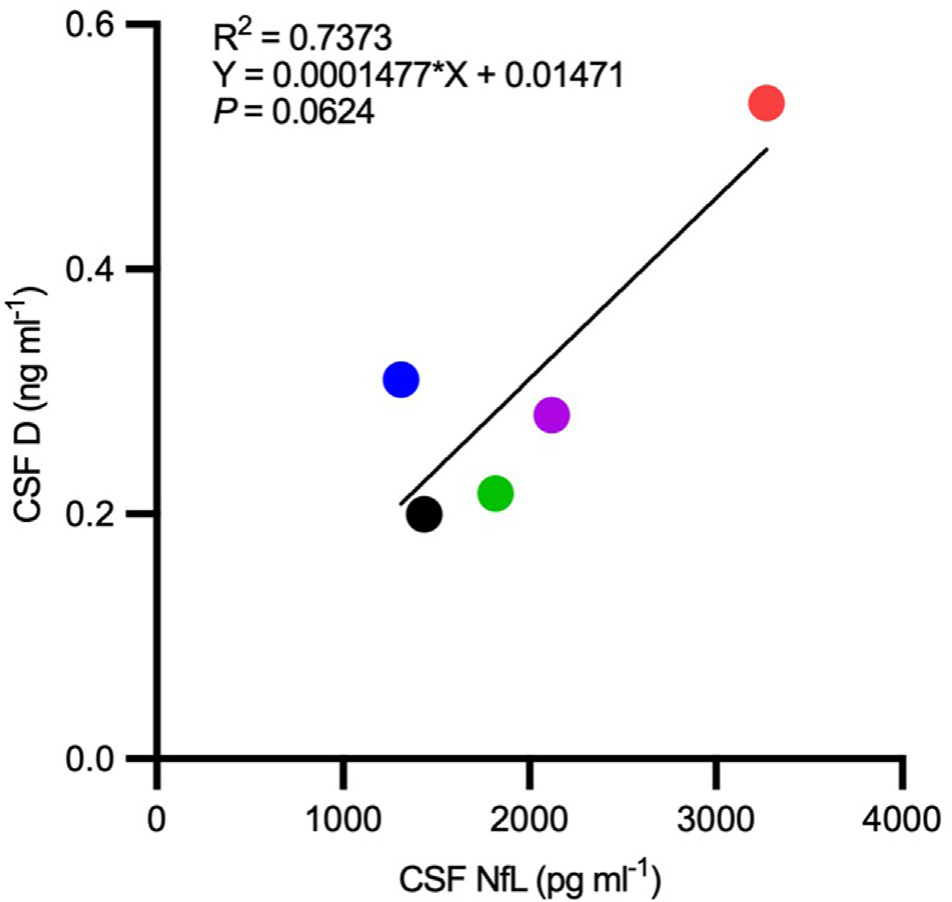
**Correlation between cerebrospinal fluid (CSF) dasatinib (D) *versus* neurofilament light chain (NfL) levels.** Post-treatment dasatinib (D; ng/ml) level correlation with baseline cerebrospinal fluid neurofilament light chain (NfL; pg/ml) derived from simple linear regression. R^2^ ​= ​0.7373; *P* ​= ​0.0624.

Pathogenic deposition of Aβ and tau protein in the brain represent the hallmark pathologies of AD. CSF levels of specific Aβ and tau protein species track with AD severity and disease progression [[23], [24], [25],[36], [37], [38], [39], [40], [41]]. Mass spectrometry was used to quantify levels, fragments and/or post-translationally modified Aβ and tau in CSF. Baseline and post-treatment Aβ42 and Aβ40 levels and the Aβ42:40 ratios were calculated for each participant (Supplementary Fig. 1a–d) and descriptive statistics are presented in Supplementary Table 3. No statistically significant changes were observed. Similarly, baseline and post-treatment CSF levels of phosphorylated tau and corresponding endogenous peptides were measured (pT153, tau 151–155, pT181, tau 181–190, pS199, pS202, pT205, pS208, tau 195–210, pT217, tau 212–221, pT231, tau 226–230). The phosphorylated tau occupancy at different tau residues (pT111/T111, pT153/T153, pT181/T181, pS199/S199, pT205/T205, pS208/S208, pT217/T217, pT231/T231), as well as the levels of microtubule binding region (MTBR) for tau 212–221 (MTBR-tau212-221) and tau 243–254 (MTBR-tau243-254, an indicator of tau neurofibrillary tangles [40]) did not change significantly during the study period (Supplementary Fig. 2 a-j and Supplementary Table 3). We also evaluated the association between post-treatment levels of D in the CSF and markers of neuropathology that reflect disease severity and may inform on BBB integrity, thus brain exposure to senolytics. While there was no correlation between D and measures of Aβ or tau analytes, we did however observe a trend (R^2^ ​= ​0.7373; *P* ​= ​0.0624) for increased uptake of D into the CSF by individuals with higher baseline CSF NfL concentrations, a marker of neurodegeneration [42] (Fig. 2).

### Metabolic analysis in urine samples

Previous work has suggested that urinary metabolites may serve as reliable biomarkers for aging [43], and AD [44]. Mass spectrometry detected 13 of the 17 common urinary amino acid and related metabolites at baseline and post-treatment. Baseline to post-treatment paired samples *t*-tests showed no statistically significant differences in any metabolites between study visits (Supplementary Table 4). Sulpiride, glutamine, glutamic acid, and nicotinic acid were excluded from analyses as urinary concentration of these metabolites were below the limit of quantitation (0.1 ​μM).

### Lipidomics analysis in plasma and CSF

**Fig. 3:**
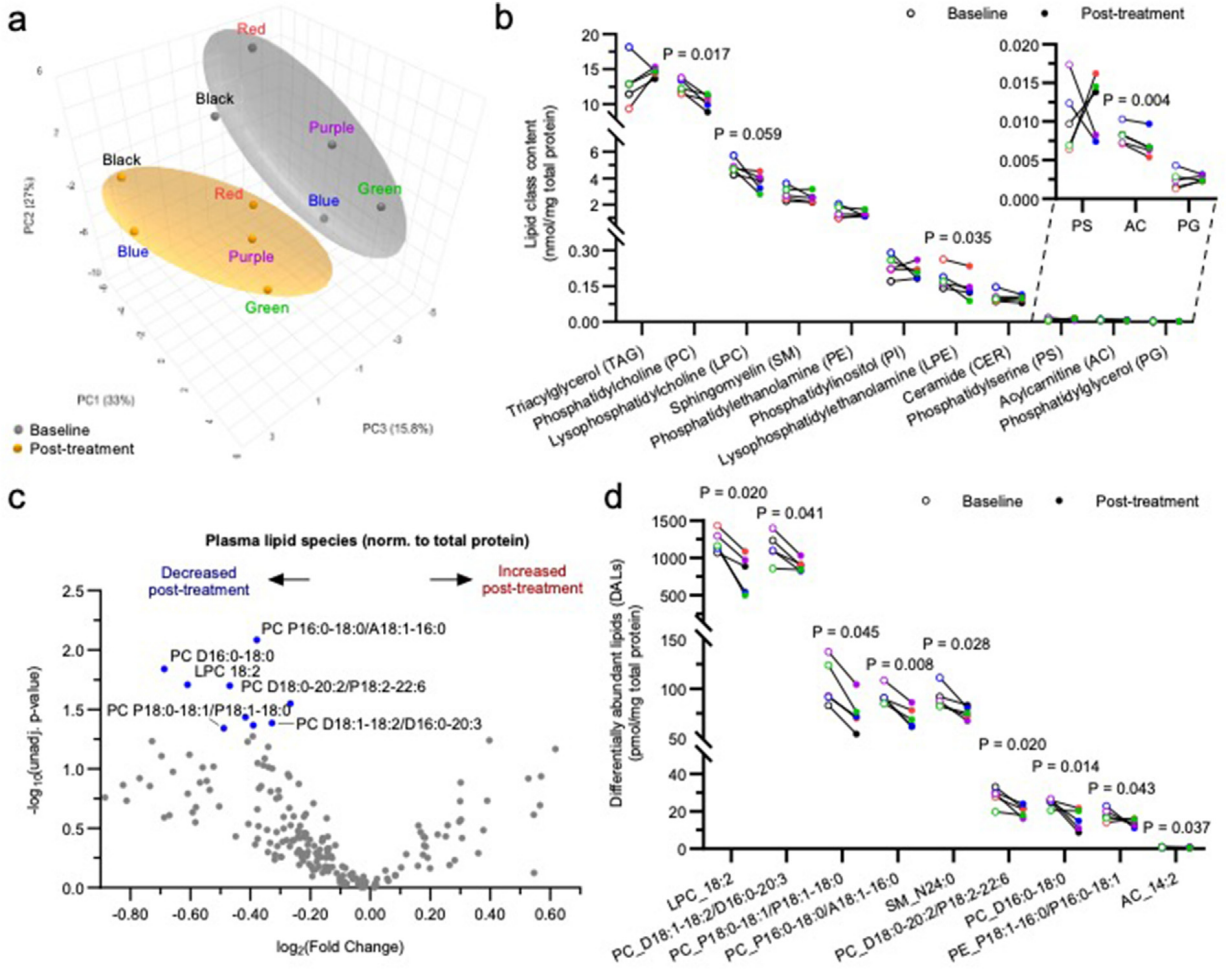
**Effects of dasatinib plus quercetin (D ​+ ​Q) treatment on the circulating plasma lipidome normalized to total protein concentration.** a–d, Effects of dasatinib plus quercetin (D ​+ ​Q) treatment on the circulating plasma lipidome normalized to total protein content. Plasma lipidome was assessed using multidimensional mass spectrometry-based shotgun lipidomics. a, MetaboAnalyst unsupervised PCA plot reducing all plasma lipid species data into three dimensions. Baseline and post-treatment groups are color-coded in gray and orange respectively, subjects are color coded to match color code assignments across all figures. b, All 11 lipid classes assessed in plasma samples. Paired samples are connected with a line. c, Volcano plot comparing all 194 plasma lipid species at baseline and post-treatment. d, Plot of the nine differentially abundant lipids (DALs) lipid species significantly decreased from baseline to post-treatment. Paired samples are connected with a line, each color represents a different subject (N ​= ​5). Only *P* ​< ​0.1 are shown.

Lipidomics is an emerging area with promising biomarker potential, as lipid changes in plasma and CSF have been previously associated with perturbed lipid metabolism linked to AD [20]. MetaboAnalyst unsupervised metadata analysis on plasma lipidomics data using all 194 detected lipid species revealed that among all factors assessed (pre/post senolytic treatment, biological sex, subject, age, and pre/post MoCA scores), biological sex had the strongest impact on the circulating lipidome, followed by senolytic treatment (Supplementary Fig. 3a). Total protein content in both plasma and CSF was stable across timepoints (Supplementary Fig. 3b). Initial metadata analysis revealed that sex separation was largely driven by principal component 1 (33%) (Supplementary Fig. 3c). Subsequent analyses were performed following MetaboAnalyst paired one factor module using transformed and scaled lipid mass levels expressed relative to plasma total protein content for both time points (Supplementary Fig. 3c). Principal component analysis (PCA) 3D scatter-plotting revealed an evident separation between baseline and post-treatment sample clusters, suggesting that senolytic treatment had an impact on the circulating lipidome as a whole (Fig. 3a). This separation was primarily driven by principal component 2 (27%) and principal component 3 (15.8 ​%) (Fig. 3a).

Paired comparisons between baseline and post-treatment plasma samples at the lipid class level revealed that out of the 11 lipid classes analyzed, three classes were significantly decreased post-treatment (Fig. 3b). These included phosphatidylcholine (PC), the most abundant phospholipid in circulation and major constituent of lipoprotein membranes, which decreased post-treatment by 17% (*P* ​= ​0.017), a biologically relevant amount considering that circulating PC levels are tightly regulated; lysophosphatidylethanolamine (LPE), a cleavage product of the second most abundant phospholipid (PE), which decreased by 22% (*P* ​= ​0.035); and acylcarnitine an intermediate of fatty acid oxidation present at very low levels in circulation that was decreased by 16% (*P* ​= ​0.004). The low acylcarnitine levels in circulation is consistent with previous reports and reflects the few cell-free mitochondria or peroxisomes in plasma, the sites where acylcarnitines are produced and reside. Lysophosphatidylcholine (LPC), the most abundant lysolipid in the circulation associated with inflammation, apoptosis, oxidative stress, and atherosclerosis [[45], [46], [47], [48], [49]], displayed a 24% decreasing trend (*P* ​= ​0.059) (Fig. 3b).

Paired comparisons between baseline and post-treatment plasma samples at the lipid species level revealed nine differentially abundant lipid species (DALs) when applying an unadjusted *P* ​< ​0.05 cut-off, all decreased post-treatment (Fig. 3c). More than half of these DALs were PC species of high, medium, or low abundance, including both diacyl and plasmalogen species (Fig. 3d). Additional DALs included the second most abundant LPC species (18:2), which was significantly reduced by 35%, and the fourth most abundant acylcarnitine species (14:2) (Fig. 3d).

**Fig. 4:**
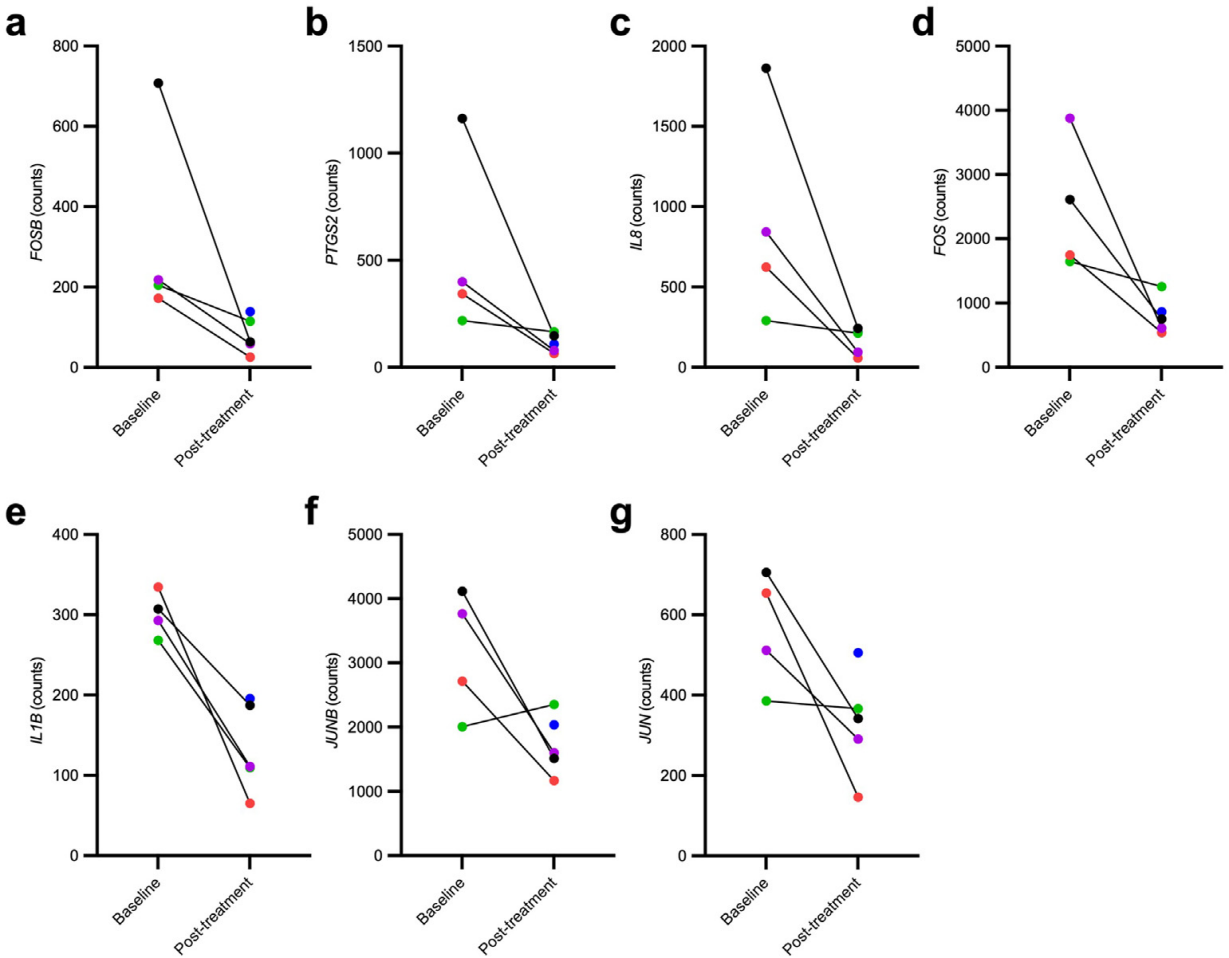
**Baseline and post-treatment significantly differentially expressedconservedtranscriptionalresponse toadversity (CTRA) gene counts in peripheral blood mononuclear cell samples measured with nanoString nCounter XT CodeSet custom CTRA gene expression panel.** a–g, Effects of dasatinib plus quercetin (D ​+ ​Q) on the expression of Conserved Transcriptional Response to Adversity (CTRA) gene counts measured in peripheral blood mononuclear cell (PBMC) samples. Seven inflammatory genes were significantly decreased post-treatment. Log2 fold-change (B-statistic): a, *FOSB*, −2.176 counts (−0.963); b, *PTGS2*, -1.980 counts (−1.395); c, *IL-8*, -2.439 counts (−1.436) (d) *FOS*, −1.584 counts (−1.849); (e) *IL-1B*, −1.386 counts (−2.082), (f) *JUNB*, −0.859 counts (−3.605) (g) *JUN*, −0.969 counts (−3.808). Baseline to post-treatment changes were assessed using a moderated *t*-test by Robust Empirical Bayes analysis in limma, *P* ​< ​0.05, N ​= ​2–4, color coded by participant. Paired baseline and post-treatment measures existed for all but one of the participants (blue) for whom only a post-treatment PBMC sample was collected. No correction for multiple comparisons was made due to small sample size.

It is important to note that if samples are normalized to plasma volume, no separation is observed by PCA (Supplementary Fig. 4a), presumably due to the higher intrinsic variability/noise associated with normalizing analytic results to sample volume. It is also worth mentioning that when normalized to plasma volume, only one class was significantly altered by treatment: triacylglyceride (TAG), which was increased post-treatment by 23% (Supplementary Fig. 4b; *P* ​= ​0.022). These results are consistent with those obtained via lipid panel lab testing, which are also expressed by volume, where a 28% post-treatment increasing trend was observed (*P* ​= ​0.064) as previously reported [16]. Additional analysis at the lipid subclass level revealed that long-chain fatty acyl-containing TAGs were significantly increased (Supplementary Fig. 4c). Moreover, consistent with the above-described protein content-based results, LPC also tended to decrease when normalizing to volume (Supplementary Fig. 4b; *P* ​= ​0.066). Lipid subclass analyses revealed that long-chain fatty acyl-containing LPCs tended to decrease (Supplementary Fig. 4d). Finally, the vast majority of the volume-normalized DALs (4 out of 5) were TAG species, which increased post-treatment (Fig. 4e–f).

Lastly, unsupervised dimensionality reduction (PCA) of CSF lipidomics data using all 79 detected lipid species normalized to total protein content revealed no separation between baseline and post-treatment samples (Supplementary Fig. 5a), implying a lack of global senolytic effect at the whole CSF lipidome level. At the lipid class level, paired comparisons revealed that none of the nine lipid classes assessed in the CSF were significantly altered post-treatment. At the lipid species level, paired comparisons revealed five DALs when applying an unadjusted *P* ​< ​0.05 cut-off, including the second most abundant LPC species (16:1) in the CSF that was reduced by 43% post-treatment (Supplementary Fig. 5c and d; *P* ​= ​0.014), the largest effect observed by magnitude. Notably, this same LPC species came up as the most important feature on a partial least squares-discriminant analysis (PLS-DA), a supervised dimensionality reduction method that was able to largely separate baseline and post-treatment samples (Supplementary Fig. 5b). The other four DALs that were significantly increased post-treatment included two PC species that were increased by 16% (D16:0–16:0, the second most abundant PC species in CSF) and 21% (D16:1–16:0/D14:1–18:0, a medium abundant PC species) post-treatment (Supplementary Fig. 5c and d). When CSF lipidomics data were normalized to volume content, none of the lipid classes nor species were significantly altered. Only one species (LPC 16:1) trended downward post-treatment (41% decrease, *P* ​= ​0.080). The decrease of LPC 16:1 in the CSF, which reached significance when normalized to protein content as mentioned above, was reminiscent and consistent with the decreases observed with other lysophospholipid species in plasma.

### Effects of senolytic therapy on a transcriptomic stress profile

Chronic stress is a known driver of both cellular senescence [50] and the pathologies and symptomology of AD [51,52]. We measured transcriptomic changes in a chronic stress-related gene profile termed the conserved transcriptional response to adversity (CTRA) [21] in peripheral blood mononuclear cells (PBMCs). Transcriptomic analysis of RNA isolated from PBMC samples revealed baseline to post-treatment downregulation of seven of the 19 inflammatory related genes included in the CTRA transcriptomic stress profile. These included *FOSB*, *PTGS2*, *IL8*, *FOS*, *IL1β*, *JUNB*, and *JUN* (*P* ​< ​0.05; Fig. 4, Supplementary Table 5). No significant differences were seen between time points for genes within the Type I interferon or antibody synthesis categories (Supplementary Table 5).

## Discussion

Reliable methodologies to identify senescent cells, assess their clearance, and evaluate related clinical efficacy are essential for successfully translating senolytics from promising pre-clinical therapeutics to clinical applications [7][114]. This study aimed to explore a broad panel of analytes for their senolytic-associated changes between baseline and a single post-treatment timepoint, using samples collected in a parent study designed to assess the safety and brain penetrance of D ​+ ​Q. Similar to other small pilot trials, these exploratory outcomes aim to identify biomarkers that should be prioritized for follow-up in longer, appropriately controlled ADRD cohort studies to investigate efficacy [53,54]. Leveraging biofluids from the first-in-human senolytic pilot study for AD, we analyzed multiple analytes across accessible biofluids (plasma, CSF, and urine) to determine their levels, variance and potential response to senolytics.

Quantifying SASP in circulating blood using panels and assays utilized by prior D ​+ ​Q studies provides a step toward determining treatment synergies from disease-specific responses. We previously presented the baseline to post-treatment changes in plasma and CSF SASP factors measured using the Mesoscale Discovery U-Plex Biomarker Group 1 (hu) 71-plex panel (MesoScale Discovery) [16]. Here, we utilized the Facility for Geroscience Analysis lab to evaluate secreted factors in plasma, CSF, and urine samples using multiplex magnetic bead immunoassays as done in other D ​+ ​Q clinical trials [14,15]. In agreement with our initial report, the majority of protein analytes remained unchanged baseline to post-treatment. While this may be in part due to the small sample size and limited power for detecting small effects, the result demonstrates consistency between assays, and with the IPF D ​+ ​Q trial [14]. A few SASP related plasma proteins revealed nonsignificant modest decreases post D ​+ ​Q treatment (*e.g*., eotaxin, MCP-1, VEGF), which previously have been shown to be elevated in AD [55], and negatively associated with memory in MCI and AD [56]. We also observed three circulating proteins which were significantly elevated post-treatment (plasma MMP-7 and fractalkine; and CSF IL-6). Though elevation of cytokines is generally indicative of inflammation, these proteins play critical roles in necessary and beneficial immunological responses in neurodegenerative disease and senescent cell clearance. For example, upregulation of fractalkine, a chemokine that dampens the pro-inflammatory state of microglia and plays a role in adult neurogenesis, has been shown to reduce tau pathology and neurodegeneration in an animal model [57], and elevated plasma fractalkine levels were protective in a stroke population [58]. The increase in CSF IL-6 at the post-treatment visit compared to baseline is consistent with data we reported using the Mesoscale [16]. In plasma we observed a non-significant 1.44-fold increase in IL-6 at post-treatment compared to baseline, whereas D ​+ ​Q reduced levels of plasma IL-6 in a population with diabetic kidney disease [15]. While the same laboratory measured IL-6 in both trials, we note important differences in the trial duration (12 weeks vs 3 days) and sample collection time after the final dose (3 ​h versus 11 days) which highlights the need for harmonizing senolytic trials to better infer outcomes. Without a control group and longer trial duration, we cannot infer what caused the changes in circulating proteins. These changes may reflect an increase in inflammation as occurs during the natural disease course, and/or may possibly be indicative of an acute inflammatory response induced by D ​+ ​Q senescent cell ablation or “senolysis”. An on-going phase 2 trial will measure SASP related factors at multiple time points after the final D ​+ ​Q dose, which may help differentiate acute versus chronic effects of treatment and clarify the discrepant results between pilot trials (ClinicalTrials.gov: NCT04685590) [12].

We also identified biofluid specific changes in SASP analytes during the study. For example, IL-6 levels were significantly different between baseline and post-treatment in the CSF only. If a change in IL-6 was used to power a future trial, CSF data indicate that a similar study conducted with nine participants could yield statistically significant results. This sample size would allow the detection of a 1.47 ​pg ​ml^−1^ difference with 80% power given the variability observed here. However, running the same power analyses using plasma IL-6 levels, 55 participants would be needed to achieve 80% power, with a significance level of 0.05 and an effect size of approximately 1.00. As such, the data generated in this study provide valuable information on anticipated analyte response to senolytics that can help guide the design and biofluid-specific outcome measures to consider in future senolytic trials. We encourage the use of this data, as appropriate, to inform study design, facilitate hypothesis generation, and assist in determining sample sizes based on specific interests and study needs, while considering the limitations of the small study size and the potential for false positives or negatives. In support of this, as demonstrated by Supplementary Table 2, we demonstrate the value of the generation of a multi-analyte disease (neurodegenerative disease) and/or treatment (senolytic, specifically D ​+ ​Q) specific SASP panel composite score to aid the interpretation of SASP relevant changes. For example, based on our data, determining a post-senolytic change in a single SASP marker, such as IL-6, would require more than 7 times the number of participants necessary to observe a change in a composite score made up of our 13 analytes. Further, our panel generated from markers determined significant after D ​+ ​Q treatment in an AD population, is more sensitive to changes than what would be expected in a panel generated from analytes determined modifiable in another disease states (i.e. diabetic kidney disease) measured at a time point more temporally distant from final senolytic dosage. For example, even in cases where moderate (0.6) attenuation might be anticipated, 80% power can still be achieved with our 13-analyte composite score at substantially lower levels (N ​= ​25/arm) than would be predicted based on the changes observed in SASP markers measured in the previous senolytic trial (N ​= ​140/arm) [35]. Our data highlight the utility of composite scores for use in future studies to substantially aid the analysis and interpretation of changes observed in plasma inflammatory markers measured as part of future senolytic trials while increasing power and effect size and reducing required sample sizes.

In parallel to studying molecules that may directly respond to senolytics, we simultaneously quantified AD biomarkers which may serve as surrogate measures of efficacy as they are predicted to be downstream of senescent cell clearance. CSF levels of Aβ and phosphorylated tau, as assessed by immunoassays, correlate with AD disease state and neurodegenerative pathology, but more disease specific information can be gleaned from assessment of the specific post-translational modifications of tau [37,59]. To obtain a comprehensive analysis of ADRD biomarkers within our sample population before and after senolytic therapy, we analyzed levels of Aβ and phosphorylation epitopes on tau protein and its fragments currently most predictive of amyloid plaques and NFT pathology [24,25,36,39,60]. The Aβ and tau biomarkers in our small open-label trial measured by mass spectrometry were unchanged from baseline to post-treatment. These results are consistent with those data presented in the initial SToMP-AD pilot trial publication [16], which were measured with the Simoa HD-X analyzer (Quanterix, Lexington, MA) and Fujirebio G1200 (Malvern, PA, Lumipulse assay), further supporting the reliability of the results. Throughout the disease course, Aβ [38] and tau [41] biomarkers gradually change over many years to decades, but levels (particularly of Aβ) are dynamic, with previous studies demonstrating that alterations in production and clearance are observable in response to a number of interventions even within a short period of time (< 4 weeks) [61,62]. Unless plaque and tangle pathologies are changing rapidly, significant alterations in these biomarkers may not be expected in the 12-week intermittent treatment period of the pilot study. The larger (N ​= ​48), placebo-controlled D ​+ ​Q trial in AD (NCT04685590), with a one-year post-treatment follow-up period is better positioned to inform on disease modification and/or stability [12].

With advancing neurodegenerative disease, BBB integrity becomes compromised [63]. Understanding the implications of BBB disruption on the penetrance, uptake, and metabolism of senolytic compounds is important to consider [114]. Elevations of NfL in serum have been linked with loss of BBB integrity in multiple sclerosis [64], but the degree to which CSF NfL is predictive of BBB integrity in AD is unclear [65,66]. As a structural protein in neuronal axons, presence of NfL in CSF is most often associated with higher levels of neurodegeneration. The observed trend for increased levels of D in CSF in participants with higher levels of baseline CSF NfL may indicate greater drug penetrance due to BBB breakdown or more advanced disease stage. However, we are cautious with this interpretation as the correlation is largely driven by one subject with high NfL levels. Future studies with a larger sample size will be necessary to determine if there is a true correlation between the concentration of NfL or other ADRD biomarkers with the concentration of D in the CNS. Nevertheless, factors relevant to AD severity and neurodegenerative disease progression are important to consider in regard to therapeutic dosing and efficacy within and across individuals [114]. Well-designed pharmacokinetic/pharmacodynamic studies will be necessary to fully understand the distribution and metabolism of senolytic compounds in healthy controls versus those with neurodegenerative disease. Additionally, the inclusion of measures more directly informative about BBB integrity may be considered in future trials, including dynamic contrast enhanced MRI (DCA-MRI) imaging analysis [67,68] or CSF biomarkers such as PDGFRβ [69].

Urinary metabolite profiles have been linked with AD and proposed as potential biomarkers for mild cognitive impairment and AD [70]. Recent studies from our group indicate the utility of urine metabolomics to understand complex diseases [71]. In this study, however, urinary metabolomics were unchanged. Although larger placebo-controlled studies are necessary, these preliminary results are encouraging as no changes in urinary metabolites associated with adverse events or AD pathogenesis were observed. Further, there are other urinary protein biomarkers (i.e. α-klotho and osteoactivin (GPNMB)) that may have great value in future clinical senolytic trials, which have previously demonstrated utility as predictive markers of biological age [72] and senescent cell load [73]. Both have successfully been altered by senolytic intervention in previous preclinical and clinical populations [73,74]. Due to the limited sample volumes available in the study, not all analytes of interest could be measured. Nevertheless, assessment of these markers should be considered in the ongoing placebo-controlled SToMP-AD study (NCT04685590), and may be of interest to others utilizing senolytics in the context of age-related diseases.

Due to the high lipid content of the brain and the critical role that lipids play in the integrity and function of cell membranes, lipidomic measurements offer important insights to brain health and disease processes [75]. Recent work indicates that lipid metabolism and homeostasis becomes dysregulated with advancing neurodegeneration [76,77] and general lipidomic dysregulation in AD [20,78] highlighting the potential utility of lipid measurements to be used as AD biomarkers. Further, lipidomic dysregulation has been posed as a driver of cellular senescence and associated inflammation [79,80], which make understanding the lipidome in AD important from the perspective of both biomarker potential and an AD driving insult to be targeted therapeutically. Lipid abundance relative to total protein content is the most commonly used and preferred normalization method for lipidomics studies [28,81,82], particularly for the assessment of lipids in plasma, where virtually all lipids are bound to protein transporters (*e.g*., albumin and lipoproteins). However, in clinical settings lipid levels are often normalized to sample volumes. Therefore, we conducted the analyses both ways to determine if both normalization methods provided similar results when assessing response to senolytics. Despite the relatively short duration of senolytic treatment in this study, unsupervised lipidomics analysis revealed global post-treatment effects on the circulating lipidome, when expressed relative to protein content, that were of potential biological relevance. Although the senolytic treatment may have affected the circulating lipidome, the effect was relatively mild as it was largely washed out when less stringent normalized methods were applied (e.g., normalized to sample volume as in clinic settings). When lipid levels were normalized to total protein, senolytic treatment led to significant decreases in total LPE content and a major LPC species in plasma. The reduction in circulating lysophospholipids observed following D ​+ ​Q treatment is suggestive of reduced inflammatory pathways, as LPC is strongly associated with pro-inflammatory signaling (reviewed in: [83,84]), and consistent with the obtained CTRA transcriptomic results. Additionally, we found an increase in circulating TAG when lipid concentrations were expressed relative to plasma volume. In the SToMP-AD pilot study, we previously reported a statistically significant, though modest and not clinically significant, increase in total cholesterol, as well as trends toward higher post-treatment total triglyceride levels and LDL cholesterol in the clinical lipid panel (*P* ​= ​0.064; Supplementary Table 1 of the parent trial results [16]), providing complimentary evidence for increased plasma neutral lipid content. We also observed decreases per protein content of total PC, the most abundant phospholipid on lipoprotein particle membranes. Taken together, TAG, cholesterol and and PC data suggest that senolytic therapy may illicit modest biological modifications of circulating lipoprotein profiles. The observed trends in lipid content are consistent with slight increases in LDL particle numbers, which could confer increased risk for atherosclerotic cardiovascular disease [85]. For this reason, we advise monitoring LDL-cholesterol, TAG, and ApoB levels (proxy for LDL particle numbers) in ongoing and future senolytic trials. Finally, the observed decreases in circulating acylcarnitines point toward a putative effect of senolytics on energy metabolism, specifically on fatty acid oxidation. Alternatively, it is possible that the changes observed in this small open-label pilot are due to normal ADRD related disease progression, as reductions in LPE [86], PC [87,88], and type-specific acylcarnities [89,90], have been associated with AD progression. Regardless of cause, these lipidomic data are examples of perhaps the most robust alterations pre-to post-senolytic treatment of all measures considered herein. Further validation is required to determine if these lipid outcomes may be utilized as sensitive biomarker indicators for response to senolytic in future ADRD trials.

Even though the short senolytic treatment did not have global effects on the CSF lipidome, it led to decreases in a specific lysophospholipid species (LPC 16:1). These results may be biologically relevant given the high abundance of this LPC species that is consistent with the decreases in lysophospholipids observed in circulation and the expected anti-inflammatory effects of senolytics. Taken together, these results place lipids as particularly sensitive and clinically valuable markers, and are consistent with preclinical studies [91,92] (for review: [93,94]). Future studies may continue evaluating wide ranges of lipid class species, as done here, to better understand the lipidome response to senolytic therapy. Complimentarily, we also propose the development of targeted lipid panels and analyses using those we identified to be most responsive to senolytics. The lipid classes and species identified here are appealing candidates to validate in independent trials.

Senolytics have been proposed as a potential therapeutic for chronic-stress induced memory deficits [50]. The CTRA represents a transcriptomic profile activated by chronic stress that is measured in circulating PBMCs [95]. Specifically, the CTRA transcriptomic pattern can be defined by relative upregulation of 19 inflammatory genes, and relative downregulation of 31 type-1 interferon response genes and three antibody synthesis genes [21,95] as displayed in Supplementary Table 5. The CTRA has been proposed as a potential predictive biomarker for disease risk and pathogenesis related to diseases impacted by inflammatory and interferon response alterations including cancer and heart disease [96], and may have utility as an indicator of AD progression [21]. A recent study showed that CTRA gene expression was higher in MCI and cognitively normal participants with lower eudemonic well-being, based on measures of perceived stress and well-being [97]. Given that senescence is a chronic cellular stress response, we were interested in assessing the utility of the CTRA as an outcome measure relevant to senescence and senolytic response in AD. Our study identified a baseline to post-treatment reduction in *FOSB, PTGS2, IL8, FOS, IL1β, JUNB*, and *JUN* expression in PBMCs. Elevated levels of each of these transcripts have been associated with senescence and SASP secretion [[98], [99], [100], [101], [102], [103], [104]], with our results indicating downregulation of pathways involved in inflammation and cell fate decisions [105,106] to provide suggestive evidence for senolytic target engagement in peripheral blood cells in this pilot study. The consistent decrease in expression across all seven of these inflammatory markers is encouraging from a therapeutic standpoint as chronic peripheral inflammation [107,108], and even psychological stress [109], is associated with AD. Our preclinical study of senolytics for AD previously reported an upregulation in *IL1β* in the brain associated with neurofibrillary tangles that decreased with D ​+ ​Q [2]. An independent preclinical trial also reported downregulation of *IL1β* in the brain in response to D ​+ ​Q [4]. Recent publications indicate that treatments with D ​+ ​Q in animal models reduce markers of inflammation associated with the pro-inflammatory SASP, including IL1β (as measured in intestinal and adipose tissue in mouse models) [110,111], which was also reduced in this trial. These prior studies demonstrate replicability of our finding, and provide evidence that measuring *IL1β* gene expression in PBMCs may be an appropriate, sensitive, biomarker indicative of a senolytic effect.

We also observed non-significant increases in *IFI27L1, IFITM1*, and *IFITM4P* type-1 interferon response genes. Given that these genes are typically down-regulated in CTRA, the data provide additional support that senolytics may be positively impacting this chronic stress pathway. While the gene expression findings in our study suggest that senolytics may impact the CTRA, significant changes would not have survived multiple comparisons correction; our preliminary findings require further replication in studies designed to assess this endpoint. Additional work is needed to understand the CTRA transcriptomic profile in AD, its utility as a biomarker, and the implications of changes in these markers in response to senolytic therapy. Our team is currently working on establishing baseline measures in AD, and will assess the senolytic-associated change in the larger Phase 2 SToMP-AD trial (NCT04685590) [12]. Other trials of particular interest in validating changes in CTRA are those focused on D ​+ ​Q in treatment resistant depression (NCT05838560) [112].

In summary, these exploratory analyses identified biofluid-specific analytes that were differentially abundant between baseline and after a 12-week intermittent D ​+ ​Q senolytic treatment. These include increased plasma fractalkine, MMP-7 and CSF IL-6, and reduced circulating PC, LPE, acylcarnitine lipid species in plasma and transcriptomic markers of the CTRA in PBMCs (*FOSB, PTGS2, IL8, FOS, IL1β, JUNB*, and *JUN*). Though significant changes in natural disease course are not expected over the relatively short treatment period of 12 weeks, we cannot confidently interpret a senolytic response without a placebo arm. We acknowledge that the small sample size and lack of a placebo-controlled arm significantly limit the strength of conclusions that can be drawn from this dataset. Nevertheless, the significant changes observed in analytes derived from lipidomics and stress-related transcriptomic assays, two areas not extensively explored in AD or senescence-focused trials, represent novel methodologies to be further developed for senolytic trials in ADRD. In parallel to identifying analytes that may directly respond to senolytics, we simultaneously quantified AD biomarkers which may serve as surrogate measures of efficacy as they are predicted to be downstream of senescent cell clearance. We observed stable expression of all Aβ and tau proteins measured, but without a longer trial duration and placebo arm, we are unable to interpret disease stability or modification. There is immediate opportunity to confirm our biomarker findings in on-going/recently completed phase 1 D ​+ ​Q trials in AD (ALSENLITE: NCT04785300; STAMINA: NCT05422885 [113]), one for treatment-resistant depression (NCT05838560) and a double-blind, placebo-controlled phase 2 study (SToMP-AD: NCT04685590). A recent publication from the STAMINA study (Senolytics to Alleviate Mobility Issues and Neurological Impairments in Aging) by our collaborators reported outcomes from twelve adults at risk of developing AD based two significant predictors of AD risk, slow walking speed and a diagnosis of MCI, after they received a dosing regimen of intermittent D ​+ ​Q (100 ​mg of D with 1250 ​mg of Q daily) for 12 weeks [113]. The results of this open label study supported the previously reported favorable safety profile of D ​+ ​Q in an AD relevant population, while additionally demonstrating a significant inverse relationship between reduced TNF-α levels with higher MoCA scores after D ​+ ​Q treatment, providing evidence for potential functional benefits of senolytic therapy in an AD relevant population, which will need to be further evaluated in larger, placebo-controlled cohorts. Moreover, the inclusion of a placebo control group, collection and analysis of samples at multiple pre-and post-treatment time points, and longer trial duration in the on-going phase 2 SToMP-AD trial has potential to inform on pathways and mechanisms by which D ​+ ​Q may elicit a biologically relevant effect in persons with AD, distinguish natural AD course from a senolytic effect, evaluate re-test variability, and inform senolytic target engagement and therapeutic efficacy. As such, the results presented here provide valuable insights into sample collection and assay feasibility, and an initial step toward designing future experiments and developing biomarker panels that reflect senescent cell burden and clearance in ADRD clinical trials.

## Data availability statement

The minimum dataset necessary to interpret, verify, and extend the research presented in this article will be available upon request to the corresponding author. The trial was registered on ClinicalTrials.gov: NCT04063124, the full study protocol [22] and primary and secondary aims of the study [16] have been previously published.

## Author contributions

M.M.G. and M.E.O. conceived the project, acquired funding, analyzed and interpreted data, and edited and submitted the manuscript. V.R.G. recruited study participants, analyzed samples, conducted experiments related to CTRA analyses, collected and analyzed data, generated graphs and tables, and drafted the manuscript. J.P.P. performed assays and statistical analyses relevant to lipidomics data. J.M., N.B., and Y.H. contributed to the HPLC-MS/MS data acquisition, and interpretation. T.F.K. and J.J.M., collected biofluid samples. P.X. and B.Z. performed statistical analyses and interpreted biofluid data. A.S. and N.R. conducted urinary metabolite experiments and contributed to data acquisition and interpretation. K.S. provided oversight and support for the urinary metabolite studies and contributed to urinary metabolite data acquisition and interpretation. J.M.E.N., A.X., and T.T. contributed to biofluid analyses and interpretation. M.L.C. performed HPLC-MS/MS study design, oversight, and analyses. A.S. and N.M. provided medical oversight of the trial. S.C., R.C.P., J.L.K., and R.J.B. contributed to data interpretation. S.H. developed methodology for composite score sample size and power statistical analyses. D.M. and S.J. performed sample size and power analysis calculations. All authors edited and approved the final manuscript.

## Declaration of competing interest

The authors declare the following financial interests/personal relationships which may be considered as potential competing interests: Ronald C. Petersen reports a relationship with University of Oxford, UpToDate, and Medscape that includes equity or stocks. Ronald C. Petersen reports personal stock in AbbVie. reports personal fees from Roche, Genetech, Eli Lilly, and Nestle, and no personal fees from Eisai, outside of the submitted work. Suzanne Craft reports a relationship with TD3 Therapeutics and Neurodegenerative Consortium that includes board membership. Suzanne Craft reports a relationship with vTv Therapeutics, Cylcerion, T3D Therapeutics, and Cognito Therapeutics, outside the submitted work that includes equity or stock. Randall J. Bateman reports a relationship with C2N Diagnostics and receives income from serving on the scientific advisory board as a co-founder. Randall J. Bateman has received research funding from Avid Radiopharmaceuticals, Janssen, Roche/Genentech, Eli Lilly, Eisai, Biogen, AbbVie, Bristol Myers Squibb, and Novartis. Mitzi M. Gonzales reports personal stock in Abbvie. James L. Kirkland and Tamara Tchkonia are co-investigators on a patent Treating Cognitive Decline and Other Neurodegenerative Conditions by Selectively Removing Senescent Cells from Neurological Tissue and a patent for Treating Cognitive Decline and Other Neurodegenerative Conditions by Selectively Removing Senescent Cells from Neurological Tissue that are held by Mayo Clinic with royalties paid to Mayo Clinic by Unity Biotechnologies. Dallin Mason, Samuel Johnson, and Suzanne Hendrix are employees of Pentara Corporation which provides consulting to over 30 pharmaceutical, biotech, non-profit, and academic groups doing clinical research in neurodegenerative disorders. Suzanne Hendrix is the founder, owner, and CEO of Pentara Corporation. Washington University has equity ownership interest in C2N Diagnostics and receives royalty income based on technology (Stable Isotope Labeling Kinetics, Blood Plasma Assay, and Methods of Diagnosing AD with Phosphorylation Changes) licensed by Washington University to C2N Diagnostics. Miranda Orr has patent Biosignature and Therapeutic Approach for Neuronal Senescence pending. If there are other authors, they declare that they have no known competing financial interests or personal relationships that could have appeared to influence the work reported in this paper.
