# Supplementary material for: Evaluation of exploratory fluid biomarkers from a phase 1 senolytic trial in mild Alzheimer’s disease
Source: Neurotherapeutics. 2025 Apr 23;22(4):e00591. doi: 10.1016/j.neurot.2025.e00591 (PMC12418413; doi:10.1016/j.neurot.2025.e00591)
Supplement: Multimedia component 1 [file mmc1.docx]

Supplementary Table 1. Plasma, cerebrospinal fluid and urine cytokines, chemokines, and proteinases, measured by multiplex immunoassay protein panels

| Protein | Baseline  Mean (SD) | Post-Treatment Mean (SD) | Fold Change | T-statistic(df),  *P* value,  95% CI | Baseline vs. Post-treatment Correlation coefficient(df),  *P* value |
| --- | --- | --- | --- | --- | --- |
| Plasma (pg ml^-1^) | | | | | |
| Adiponectin | 1.86e^+7^ (3.94e^+6^) | 1.80e^+7^ (3.50e^+6^) | 0.96 | t(4)= 1.726,  *P*= 0.159,  -1.76e^+7^ to 4.10e^+5^ | *r*(3)= 0.979,  *P*= 0.0035, |
| Cystatin C | 1.00e^+6^ (1.55e^+5^) | 9.60e^+5^ (1.4e^+5^) | 0.96 | t(4)= 1.114,  *P*= 0.328,  -1.44e^+5^ to 61357 | *r*(3)= 0.849  *P*= 0.0688 |
| Eotaxin | 186 (41.9) | 151 (36.3) | 0.81 | t(4)= 2.309,  *P*= 0.082,  -76.78 to 7.060 | *r*(3)= 0.6355  *P*= 0.2492 |
| FAS | 9799 (2575) | 11096 (4109) | 1.13 | t(4)= 1.259,  *P*= 0.277,  -1562 to 4154 | *rs*(3)= 0.900  *P*= 0.0833 |
| Fractalkine | 959 (584) | 1586 (613) | 1.65 | t(2)= 34.46,  *P*= 0.001,  548.90 to 705.60 | *r*(1)= 0.100  *P*= 0.0126 |
| GROα | 95.1 (25.1) | 73.3 (16.1) | 0.77 | t(3)= 1.404,  *P*= 0.255,  -71.39 to 27.69 | *r* (2)= -0.0994, *P*= 0.901 |
| GROβ | 386 (195) | 204  (87.7) | 0.53 | t(4)= 1.612,  *P*= 0.182,  -496.3 to 131.8 | *r*(3)= -0.534,  *P*= 0.354 |
| ICAM-1 | 8.41e^+5^ (1.81e^+5^) | 9.10e^+5^ (1.6e^+5^) | 1.08 | t(4)= 1.908,  *P*= 0.129,  -30801 to 1.66e^+5^ | *r*(3)= 0.900,  *P*= 0.0372 |
| IL-1Ra | 278 (81.6) | 341  (154) | 1.23 | t(4)= 0.9284,  *P*= 0.406,  -125.5 to 251.5 | *r*(3)= 0.293,  *P*= 0.633 |
| *IL-6 | 3.46 (1.85) | 4.97 (3.41) | 1.44 | t(4)= 2.097,  *P*= 0.104,  -0.488 to 3.500 | *r*(3)= 0.987,  *P*= 0.0018 |
| IL-8 | 3.78 (0.705) | 3.20 (0.700) | 0.85 | t(4)= 1.918,  *P*= 0.128,  -1.419 to 0.259 | *r*(3)= 0.537,  *P*= 0.351 |
| MCP-1 | 183 (32.8) | 159  (31.3) | 0.87 | t(4)= 1.535,  *P*= 0.110,  -66.80 to 19.24 | *r*(3) = 0.416,  *P*= 0.486 |
| MIP-3β | 96.5 (22.2) | 83.2 (23.8) | 0.86 | t(4)= 2.051,  *P*= 0.110,  -31.11 to 4.673 | *r*(3)= 0.806,  *P*= 0.0996 |
| MMP-2 | 4.75e+5 (94384) | 4.30e^+5^ (89063) | 0.91 | t(4)= 1.720,  *P*= 0.161,  -1.17e^+5^ to 27410 | *r*(3)= 0.802,  *P*= 0.103 |
| MMP-7 | 2998 (2000) | 3224 (2092) | 1.08 | t(4)= 2.779,  *P*= 0.0499,  0.198 to 452.90 | *r*(3)= 0.997,  *P*= 0.0002 |
| MMP-8 | 2753 (2652) | 3421 (2848) | 1.24 | t(4)= 0.9481,  *P*= 0.397,  -1289 to 2625 | *rs*(3)= 1.00  *P*= 0.0167 |
| MPO | 82463 (1.15e^+5^) | 2.74e^+5^ (5.10e^+5^) | 3.32 | t(4)= 1.083,  *P*= 0.340,  -3.00e^+5^ to 6.83e^+5^ | *rs*(3)= 0.700  *P*= 0.233 |
| PAI-1 | 20777 (6009) | 17794 (13207) | 0.86 | t(4)= 0.545,  *P*= 0.615,  -18181 to 12216 | *r*(3)= 0.382  *P*= 0.525 |
| PARC | 48069 (16843) | 49875 (16655) | 1.04 | t(4)= 2.091,  *P*= 0.105,  -591.80 to 4204 | *r*(3)= 0.993  *P*= 0.0006 |
| PDGF-AA | 1118 (967) | 338 (178) | 0.30 | t(4)= 1.611,  *P*= 0.182,  -2125 to 564.20 | *r*(3)= -0.601  *P*= 0.284 |
| PDGF-AA/BB | 294 (228) | 98.1 (53.1) | 0.33 | t(4)= 1.613,  *P*= 0.182,  -532.80 to 141.20 | *r*(3)= -0.779  *P*= 0.120 |
| RAGE | 2406 (716) | 2188 (827) | 0.91 | t(4)= 1.767,  *P*= 0.152,  -559.6 to 124.4 | *r*(3)= 0.946  *P*= 0.0148 |
| RANTES | 20671 (16131) | 5802 (3326) | 0.28 | t(4)= 1.850,  *P*= 0.138,  -37182 to 7444 | *r*(3)= -0.4814  *P*= 0.412 |
| TARC | 276 (268) | 227  (145) | 0.82 | t(3)= 0.762,  *P*= 0.502,  -251.10 to 154.10 | *rs*(2)= 1.00  *P*= 0.0833 |
| TNFR1 | 2177 (520) | 2373 (667) | 1.09 | t(4)= 1.749,  *P*= 0.155,  -115.40 to 508.50 | *r*(3)= 0.940  *P*= 0.0174 |
| uPAR | 1080 (462) | 1241 (624) | 1.15 | t(4)= 1.469,  *P*= 0.216,  -143.30 to 465.20 | *r*(3)= 0.942  *P*= 0.0168 |
| VEGF | 50.8 (13.1) | 37.6 (8.73) | 0.74 | t(4)= 2.445,  *P*= 0.071,  -28.19 to 1.789 | *r*(3)= 0.450  *P*= 0.447 |
| Cerebrospinal Fluid (pg ml^-1^) | | | | | |
| IL-1Ra | 55.7 (6.54) | 58.6 (10.5) | 1.05 | t(4)= 0.975*,*  *P*= 0.385,  -5.361 to 11.16 | *r*(3)= 0.791  *P*= 0.1112 |
| *IL-6 | 2.72 (0.678) | 3.78 (0.669) | 1.39 | t(4)= 5.265,  *P*= 0.006,  0.500 to 1.616 | *r*(3)= 0.778,  *P*= 0.122 |
| MIP-3α | 8.48 (2.58) | 9.30 (4.07) | 1.10 | t(4)= 0.420,  *P*= 0.696,  -4.602 to 6.242 | *r*(3)= 0.199  *P*= 0.748 |
| MIP-3β | 75.24 (13.45) | 78.24 (14.21) | 1.04 | t(4)= 1.056,  *P*= 0.351,  -4.888 to 10.89 | *r*(3)= 0.896,  *P*= 0.0397 |
| MPO | 121 (118) | 242  (383) | 2.00 | t(4)= 0.997*,*  *P*= 0.375,  -215.80 to 457.80 | *r*(3)= 0.900  *P*= 0.0833 |
| Sclerostin | 37.1 (33.8) | 34.8 (30.7) | 0.94 | t(4)=1.541*,*  *P*= 0.198,  -6.332 to 1.812 | *r*(3)= 1.00  *P*< 0.0001 |
| TNF-α | 0.700 (0.186) | 0.720 (0.112) | 1.03 | t(4)= 0.309*,*  *P*= 0.773,  -0.157 to 0.196 | *r*(3)= 0.651  *P*= 0.234 |
| TNFRI | 997 (187) | 998 (159) | 1.00 | t(4)= 0.513,  *P*= 0.635,  -95.00 to 138.00 | *r*(3)= 0.865  *P*= 0.0581 |
| Urine (mg ml^-1^) | | | | | |
| Creatinine | 0.918 (0.408) | 0.933 (0.552) | 1.02 | t(3)= 0.159,  *P*= 0.884,  -0.285 to 0.315 | *r*(2)= 0.967  *P*= 0.0327 |
| Urine Protein Normalized to Creatinine (mg ml^-1^ Creatinine) | | | | | |
| CRP | 3.75 (5.22) | 27.5 (48.8) | 7.33 | t(3)= 1.088,  *P*= 0.356,  -45.71 to 93.20 | *rs*(2)= 0.200  *P*= 0.9167 |
| Cystatin C | 365 (210) | 352  (212) | 0.96 | t(3)= 0.786,  *P*= 0.489,  -67.17 to 40.57 | *r*(2)= 0.987  *P*= 0.0128 |
| EGF | 120 (38.3) | 117  (40.2) | 0.98 | t(3)= 0.575,  *P*= 0.605,  -24.51 to 17.00 | *r*(2)= 0.946  *P*= 0.0541 |
| FAS | 12.8 (4.63) | 19.0 (16.4) | 1.48 | t(3)= 1.012,  *P*= 0.386,  -13.16 to 25.44 | *r*(2)= 0.800  *P*= 0. 333 |
| FIT-3L | 0.898 (0.295) | 1.97 (1.72) | 2.19 | t(3)= 1.215,  *P*= 0.311,  -1.73 to 3.86 | *r*(2)= -0.0544  *P*= 0.946 |
| Fractalkine | 29.9 (12.3) | 30.3 (19.5) | 1.01 | t(2)= 0.080,  *P*= 0.944,  -18.32 to 19.01 | *r*(1)= 0.991  *P*= 0.0868 |
| GDF-15 | 78.2 (64.6) | 66.2 (49.9) | 0.85 | t(3)= 1.561,  *P*= 0.216,  -36.25 to 12.39 | *r*(2)= 0.997  *P*= 0.003 |
| GROα | 0.995 (0.534) | 3.26 (3.73) | 3.28 | t(3)= 1.224,  *P*= 0.308,  -3.62 to 8.14 | *r*(2)= 0.135  *P*= 0.865 |
| ICAM-1 | 234 (24.0) | 289  (56.9) | 1.24 | t(2)=1.937,  *P*= 0.192,  -67.56 to 178.20 | *r*(1)= 0.500  *P*= 0.667 |
| IGFBP2 | 113 (37.1) | 121  (60.0) | 1.07 | t(3)= 0.620,  *P*= 0.579,  -30.00 to 44.50 | *r*(2)= 0.995  *P*= 0.999 |
| IL-1Ra | 60.5 (24.1) | 157  (121) | 2.60 | t(2)= 1.672,  *P*= 0.237,  -151.10 to 343.30 | *r*(1)= 0.909  *P*= 0.274 |
| IL-8 | 0.829 (0.695) | 1.45 (2.19) | 1.75 | t(2)= 0.795,  *P*= 0.485,  -1.88 to 3.13 | *rs*(2)= 1.00  *P*= 0.0833 |
| MCP-1 | 2.50 (2.51) | 2.01 (1.35) | 0.80 | t(2)= 0.812,  *P*= 0.476,  -2.42 to 1.44 | *r*(2)= 0.980  *P*= 0.020 |
| MIF | 57.80 (52.70) | 72.70 (73.30) | 1.25 | t(3)= 0.609,  *P*= 0.586,  -63.01 to 92.81 | *r*(2)= 0.745  *P*= 0.555 |
| MIP-3α | 1.19 (0.887) | 5.98 (6.44) | 5.03 | t(3)= 1.455,  *P*= 0.242,  -5.68 to 15.24 | *r*(2)= -0.0854  *P*= 0.915 |
| MIP-3β | 0.369 (0.359) | 0.817 (1.07) | 2.21 | t(3)= 1.248,  *P*= 0.301,  -0.693 to 1.59 | *rs*(2)= 0.800  *P*= 0.333 |
| MMP-2 | 6.86 (1.24) | 14.30 (14.0) | 2.08 | t(2)= 0.917,  *P*= 0.456,  -27.42 to 42.27 | *rs*(1)= 0.500  *P*> .999 |
| MMP-3 | 2.76 (0.98) | 3.50 (1.56) | 1.27 | t(3)= 0.926,  *P*= 0.423,  -1.816 to 3.306 | *r*(2)= 0.262  *P*= 0.738 |
| MMP-7 | 117 (73.50) | 120 (76.40) | 1.03 | t(3)= 0.113,  *P*= 0.917,  -88.44 to 94.94 | *r*(2)= 0.705  *P*= 0.295 |
| MMP-8 | 16.1 (11.20) | 106  (166) | 6.58 | t(2)= 0.998,  *P*= 0.424,  -296.60 to 475.70 | *r*(1)= 0.911  *P*= 0.271 |
| MMP-9 | 64.0 (51.0) | 295  (512) | 4.61 | t(3)= 0.940,  *P*= 0.417,  -551.9 to 1014 | *rs*(2)= 0.800  *P*= 0.333 |
| MMP-10 | 2.69 (1.57) | 5.12 (6.76) | 1.90 | t(3)= 0.872,  *P*= 0.448,  -6.457 to 11.33 | *r*(2)= 0.799  *P*= 0.201 |
| MPO | 181 (135) | 970 (1751) | 5.36 | t(3)= 0.931,  *P*= 0.421,  -1907 to 3484 | *rs*(2)= -0.400  *P*= 0.750 |
| OPN | 8673 (5082) | 6917 (4420) | 0.80 | t(2)= 0.958,  *P*= 0.409,  -7589 to 4077 | *rs*(2)= 1.00  *P*= 0.0833 |
| PARC | 0.865 (0.717) | 1.26 (0.755) | 1.46 | t(3)= 0.925,  *P*= 0.423,  -0.958 to 1.743 | *r*(2)= 0.336  *P*= 0.664 |
| PDGF-AA | 0.470 (0.264) | 0.365 (0.151) | 0.78 | t(3)= 0.877,  *P*= 0.445,  -0.486 to 0.276 | *r*(2)= 0.439  *P*= 0.561 |
| RAGE | 211 (66.8) | 152  (24.4) | 0.72 | t(3)= 1.749,  *P*= 0.179,  -166.50 to 48.42 | *r*(2)= 0.151  *P*= 0.849 |
| TNFR-I | 15.00 (4.41) | 18.10 (7.02) | 1.21 | t(3)= 1.428,  *P*= 0.249,  -3.807 to 10.01 | *r*(2)= 0.805  *P*= 0.195 |
| TNFR-II | 23.60 (8.57) | 27.90 (6.56) | 1.18 | t(3)=2.101,  *P*= 0.127,  -2.21 to 10.81 | *r*(2)= 0.887  *P*= 0.113 |
| uPAR | 3.43 (1.30) | 4.46 (0.73) | 1.30 | t(3)= 1.624,  *P*= 0.203,  -0.995 to 3.070 | *r*(2)= 0.309  *P*= 0.691 |
| VEGF | 0.818 (0.167) | 0.835 (0.180) | 1.02 | t(3)= 0.200,  *P*= 0.854,  -0.261 to 0.296 | *r*(2)= 0.494  *P*= 0.506 |
| Note: Differential expression analysis was carried out by two-sided, paired sample t-test, 95% CI: 95 percent confidence interval for the post *versus* baseline mean difference, *P* < 0.05. No correction for multiple comparisons was made due to small sample size (N= 3-5). Proteins listed in alphabetical order; statistically significant *P* values presented in bold. *: Prespecified secondary outcome. Baseline vs. post-treatment correlations were presented as Pearson r (*r*) correlations for normally distributed data, and Spearman r (*rs*) correlations for non-parametric data. CRP = C-reactive protein, EGF = Epidermal growth factor, FAS = tumor necrosis factor receptor superfamily member 6, FIT = Fms related receptor tyrosine kinase, GDF = Growth/differentiation factor, GRO = Growth related protein, ICAM = Intercellular adhesion molecule, IGFBP = Insulin-like growth factor-binding protein-1, IL = interleukin, MCP = Monocyte chemoattractant protein, MIP = Macrophage inflammatory protein, MMP = Matrix metalloproteinases, MPO = Myeloperoxidase, PAI = Plasminogen activator inhibitor, PDGF = Platelet-derived growth factor, RAGE = Receptor for advanced glycation end products, RANTES = Regulated upon activation, normal T cell expressed and presumably secreted, TARC = Thymus- and activation-regulated chemokine, TNF = Tumor necrosis factor, uPAR = urikinase plasminogen activator surface receptor, VEGF = Vascular endothelial growth factor | | | | | |

Supplementary Table 2: Power and sample size calculations for plasma SASP factor analytes

| SASP Analyte/Panel | Power | Alpha | Effect Size | Attenuation | Attenuation Effect Size | N/Arm |
| --- | --- | --- | --- | --- | --- | --- |
| IL-1**α**, IL-1RA, IL-2, IL-6, IL-9, FGF-2, GM-CSF, MMP-9, MMP-12, MMP-2^1^ | 0.8 | 0.025 | 0.842 | 0.0 | 0.842 | 24 |
|  |  | | | 0.2 | 0.673 | 36 |
|  |  |  |  | 0.4 | 0.505 | 63 |
|  |  |  |  | 0.6 | 0.337 | 140 |
|  | | | | | | |
| IL-6, IL-10, IL-17A/F, IL-17D, IL-17E, IL-21, IL-23, IL-31, MCP-2, MIP-1α, MIP-1β, VEGF, YKL-40^2^ | 0.8 | 0.025 | 2.037 | 0.0 | 2.037 | 5 |
|  |  | | | 0.2 | 1.630 | 8 |
|  |  |  |  | 0.4 | 1.222 | 12 |
|  |  |  |  | 0.6 | 0.815 | 25 |
|  | | | | | | |
| IL-6^2^ | 0.8 | 0.025 | 0.738 | 0.0 | 0.738 | 30 |
|  |  | | | 0.2 | 0.591 | 46 |
|  |  |  |  | 0.4 | 0.443 | 81 |
|  |  |  |  | 0.6 | 0.295 | 181 |
| Note: Global Statistical Test computations based on T-scores for SASP biomarker changes observed after D+Q senolytic administration in previously published data.  ^1^Hickson *et al*., 2020; ^2^Gonzales *et al*., 2023 | | | | | | |

Supplementary Table 3: Baseline and post-treatment cerebrospinal fluid tau levels measured by mass spectrometry

| Tau Phosphorylation Ratios and Fragments | Baseline  Mean (SD) | Post-Treatment Mean (SD) | Fold Change | T-statistic(df),  *P* value,  95% CI | Baseline vs. Post-treatment Correlation coefficient(df),  *P* value |
| --- | --- | --- | --- | --- | --- |
| pT111/T111 (%) | 4.16 (0.87) | 4.26 (0.99) | 1.02 | t(1)= 2.14,  *P*= 0.278,  -5.78 to 4.12 | N/A |
| pT153/T153 (%) | 0.53 (0.30) | 0.61 (0.42) | 1.14 | t(4)= 0.354,  *P*= 0.742,  -0.51 to 0.66 | *r*(3)=0.191,  *P*= 0.759 |
| pT181/T181 (%) | 35.3 (2.34) | 34.8 (2.35) | 0.99 | t(4)= 2.042,  *P*= 0.111,  -1.17 to 0.18 | *r*(3)= 0.973,  *P=* 0.0052 |
| pS199/S199 (%) | 0.88 (0.20) | 0.85 (0.17) | 0.96 | t(4)= 0.948,  *P*= 0.397,  -0.13 to 0.07 | *r*(3)= 0.919,  *P*= 0.0276 |
| pS202/S202 (%) | 2.18 (0.58) | 2.2 (0.56) | 1.01 | t(4)= 0.198,  *P*= 0.853,  -0.20 to 0.23 | *r*(3)= 0.956,  *P*= 0.0110 |
| pT205/T205 (%) | 0.97 (0.19) | 1.02 (0.15) | 1.05 | t(4)= 1.575,  *P*= 0.190,  -0.035 to 0.13 | *r*(3)= 0.950,  *P*= 0.0131 |
| pS208/S208 (%) | 0.15 (0.03) | 0.14 (0.02) | 0.99 | t(4)= 0.363,  *P*= 0.735,  -0.015 to 0.012 | *r*(3)= 0.979,  *P*= 0.0036 |
| pT217/T217 (%) | 12.10 (1.22) | 12.03 (1.27) | 0.99 | t(4)= 0.158,  *P*= 0.882,  -1.30 to 1.16 | *r*(3)= 0.683,  *P*= 0.2041 |
| pT231/T231 (%) | 18.2 (3.83) | 16.2 (4.27) | 0.89 | t(4)= 0.796,  *P*= 0.471,  -9.27 to 5.14 | *r*(3)= -0.0265, *P*= 0.9663 |
| pT153 (ng ml^-1^) | 0.018 (0.007) | 0.024 (0.018) | 1.35 | t(4)= 0.953,  *P*= 0.395,  -0.012 to 0.024 | *r*(3)= 0.646,  *P*= 0.239 |
| tau 151-155 (ng ml^-1^) | 3.84 (1.36) | 3.94 (1.15) | 1.03 | t(4)= 0.572,  *P*= 0.598,  -0.36 to 0.55 | *r*(3)= 0.970,  *P*= 0.0061 |
| tau 181-190 (ng ml^-1^) | 3.68 (1.29) | 3.91 (1.16) | 1.06 | t(4)= 1.007,  *P*= 0.371,  -0.41 to 0.87 | *r*(3)= 0.918,  *P*= 0.0276 |
| pT181 (ng ml^-1^) | 1.32 (0.49) | 1.37 (0.42) | 1.04 | t(4)= 0.695,  *P*= 0.526,  -0.16 to 0.27 | *r*(3)= 0.937,  *P*= 0.0189 |
| pS199 (ng ml^-1^) | 0.034 (0.012) | 0.034 (0.009) | 1.00 | t(4)= 0.084,  *P*= 0.937,  -0.004 to 0.005 | *r*(3)= 0.992,  *P*= 0.0009 |
| pS202 (ng ml^-1^) | 0.081 (0.027) | 0.085 (0.016) | 1.05 | t(4)= 0.638,  *P*= 0.558,  -0.014 to 0.022 | *r*(3)= 0.900,  *P*= 0.0372 |
| pS208 (ng ml^-1^) | 0.006 (0.002) | 0.006 (0.001) | 1.02 | t(4)= 0.298,  *P*= 0.781,  -0.001 to 0.001 | *r*(3)= 0.952 ,  *P*= 0.0126 |
| pT205 (ng ml^-1^) | 0.037 (0.014) | 0.041 (0.010) | 1.08 | t(4)= 1.608,  *P*= 0.183,  -0.002 to 0.009 | *r*(3)= 0.990,  *P*= 0.0012 |
| T195-210 (ng ml^-1^) | 3.88 (1.32) | 4.02 (0.97) | 1.04 | t(4)= 0.615,  *P*= 0.572,  -0.47 to 0.74 | *r*(3)= 0.955 ,  *P*= 0.0115 |
| pT217 (ng ml^-1^) | 0.31 (0.11) | 0.32 (0.09) | 1.04 | t(4)= 0.480,  *P*= 0.657,  -0.055 to 0.079 | *r*(3)= 0.891,  *P*= 0.0423 |
| T212-221 (ng ml^-1^) | 2.56 (0.93) | 2.67 (0.69) | 1.04 | t(4)= 0.716,  *P*= 0.514,  -0.31 to 0.53 | *r*(3)= 0.960,  *P*= 0.0096 |
| pT231 (ng ml^-1^) | 0.090 (0.040) | 0.086 (0.036) | 0.96 | t(4)= 0.289,  *P*= 0.787,  -0.037 to 0.030 | *r*(3)= 0.756 ,  *P*= 0.1395 |
| T226-230 (ng ml^-1^) | 0.52 (0.23) | 0.54 (0.19) | 1.04 | t(4)= 0.461,  *P*= 0.669,  -0.109 to 0.153 | *r*(3)= 0.896 ,  *P*= 0.0394 |
| MTBR-tau212-221 (ng ml^-1^) | 0.15 (0.03) | 0.14 (0.03) | 0.95 | t(4)= 1.926,  *P*= 0.126,  -0.018 to 0.003 | *r*(3)= 0.980,  *P*= 0.0033 |
| MTBR-tau243-254 (ng ml^-1^) | 0.60 (0.14) | 0.62 (0.13) | 1.03 | t(4)= 0.814,  *P*= 0.461,  -0.040 to 0.073 | *r*(3)= 0.950 ,  *P*= 0.0135 |
| Note: Baseline to post-treatment changes were assessed using two-sided, paired sample t-tests, 95% CI: 95 percent confidence interval for the post *versus* baseline mean difference, *P* < 0.05. No correction for multiple comparisons was made due to small sample size (N = 2-5). Baseline vs. post-treatment correlations were presented as Pearson r (*r*) correlations. df = degrees of freedom, MTBR = microtubule binding region, pS = phosphorylated serine, pT = phosphorylated tau, SD = standard deviation, T = tau | | | | | |

Supplementary Table 4. Baseline and post-treatment urinary metabolites measured by mass spectrometry

| Urinary Metabolite | Baseline  Mean (SD) | Post-Treatment  Mean (SD) | Fold Change | T-statistic(df),  *P* value,  95% CI | Baseline vs.  Post-treatment Correlation coefficient(df),  *P* value |
| --- | --- | --- | --- | --- | --- |
| Creatinine (µM) | 4957 (2594) | 4686 (2502) | 0.95 | t(3)= 0.259,  *P*= 0.812  -3595 to 3053 | *r*(2)= 0.665  *P*= 0.336 |
| Analyte uM mM^-1^ Creatinine | | | | | |
| Alanine | 44.9 (31.1) | 38.7 (24.9) | 0.86 | t(3)= 0.408,  *P*= 0.711  -55.14 to 42.61 | *r*(2) = 0.416  P = 0.584 |
| Arginine | 3.77 (0.887) | 4.25 (1.90) | 1.13 | t(3)= 0.586,  *P*= 0.599  -2.118 to 3.073 | *r*(2)= 0.519  P = 0.481 |
| Cysteine | 1.22 (0.525) | 2.48 (2.81) | 2.03 | t(3)= 0.902,  *P*= 0.434  -3.191 to 5.716 | *r*(2)= 0.111  P = 0.889 |
| Glycine | 196 (278) | 181 (201) | 0.92 | t(3)= 0.385,  *P*= 0.726  -138.7 to 108.8 | *rs*(2)= 1.00  P = 0.0833 |
| Histidine | 35.1 (6.45) | 44.5 (25.2) | 1.27 | t(3)= 0.682,  *P*= 0.544  -34.61 to 53.48 | *r*(2)= -0.272  P = 0.728 |
| Isoleucine | 61.9 (60.6) | 59.6 (48.4) | 0.96 | t(3)= 0.0737,  *P* = 0.946  -101.5 to 96.90 | *r*(2)= 0.362  P = 0.638 |
| Leucine | 58.0 (60.9) | 51.1 (37.6) | 0.88 | t(3)= 0.287,  *P*= 0.793  -84.11 to 70.19 | *r*(2)= 0.605  P = 0.395 |
| Lysine | 21.6 (9.26) | 22.5 (22.9) | 1.04 | t(3)= 0.115,  *P*= 0.915  -23.79 to 25.58 | *r*(2)= 0.872  P = 0.128 |
| Methionine | 14.7 (7.39) | 9.06 (5.19) | 0.62 | t(3)= 1.295,  *P*= 0.286  -19.49 to 8.215 | *r*(2)= 0.0761  P = 0.924 |
| Pipecolate | 4.19 (4.79) | 2.84 (2.58) | 0.68 | t(3)= 1.216,  *P*= 0.311  -4.893 to 2.188 | *rs*(2)= 1.00  P = 0.0833 |
| Phenylalanine | 4.08 (3.01) | 6.21 (1.78) | 1.52 | t(3)= 1.208,  *P*= 0.314  -3.488 to 7.758 | *r*(2)= -0.0244  P = 0.976 |
| Proline | 12.6 (22.0) | 26.4 (49.3) | 2.10 | t(3)= 1.014,  *P*= 0.385  -29.54 to 57.16 | *rs*(2)= 0.400  P = 0.750 |
| Tryptophan | 11.2 (18.5) | 2.17 (4.33) | 0.19 | t(3)= 0.875,  *P*= 0.446  -41.84 to 23.80 | *r*(2) = -0.405  P = 0.164 |
| Note: Baseline to post-treatment changes were assessed using two-sided, paired sample t-tests, 95% CI: 95 percent confidence interval for the post *versus* baseline mean difference, *P* < 0.05. No correction for multiple comparisons was made due to small sample size (N = 4). Baseline vs. post-treatment correlations were presented as Pearson r (*r*) correlations for normally distributed data, and Spearman r (*rs*) correlations for non-parametric data; df = degrees of freedom, N/A indicates too few pairs were available to generate a correlation. | | | | | |

Supplementary Table 5: Baseline to post-treatment differential expression of Conserved Transcriptional Response to Adversity (CTRA) genes

| Gene | Baseline  Mean (SD) | Post-Treatment  Mean (SD) | Fold Change | *P* value, B-statistic | Baseline vs.  Post-treatment Correlation coefficient(df),  *P* value |
| --- | --- | --- | --- | --- | --- |
| Inflammatory Genes | | | | | |
| *FOS* | 2380.30 (1083.30) | 800.98 (251.87) | 0.34 | *P*= 0.006, B= -1.849 | *r*(2)= -0.460,  *P*= 0.540 |
| *FOSB* | 299.83 (207.83) | 80.956 (45.92) | 0.27 | *P*= 0.002, B= -0.964 | *rs*(2)= 0.400, *P*= 0.750 |
| *FOSL1* | BDT | BDT | N/A | N/A | N/A |
| *FOSL2* | 1548.96 (305.240) | 1350.91 (280.59) | 0.87 | *P*= 0.366, B= -5.793 | *r*(2)= 0.307,  *P*= 0.693 |
| *IL1α* | BDT | BDT | N/A | N/A | N/A |
| *IL1β* | 288.76 (39.46) | 135.82 (61.01) | 0.47 | P= 0.008, B= -2.082 | *r*(2)= -0.239,  *P*= 0.761 |
| *IL6* | BDT | BDT | N/A | N/A | N/A |
| *IL8* | 842.27 (563.01) | 166.34 (84.91) | 0.20 | *P*= 0.004, B= -1.436 | *r*(2)= 0.436,  *P*= 0.564 |
| *JUN* | 539.33 (132.03) | 333.76 (137.30) | 0.62 | *P*= 0.043, B= -3.808 | *r*(2)= -0.453,  *P*= 0.547 |
| *JUNB* | 3005.00 (873.97) | 1737.71 (436.42) | 0.58 | *P*= 0.035, B= -3.605 | *r*(2)= -0.521,  *P*= 0.479 |
| *JUND* | 801.92 (297.32) | 675.95 (141.83) | 0.84 | *P*= 0.436, B= -5.918 | *r*(2)= 0.238,  *P*= 0.762 |
| *NF*κ*B1* | 224.43 (22.98) | 219.38 (24.75) | 0.98 | *P*= 0.868, B= -6.276 | *r*(2)= -0.259,  *P*= 0.741 |
| *NF*κ*B2* | 485.25 (134.44) | 373.89 (57.52) | 0.77 | *P*= 0.180, B= -5.200 | *r*(2)= -0.0168, *P*= 0.983 |
| *PTGS1* | 202.86 (69.99) | 167.62 (58.76) | 0.83 | *P*= 0.246, B= -5.474 | *r*(2)= 0.875,  *P*= 0.125 |
| *PTGS2* | 490.51 (351.34) | 112.75 (41.66) | 0.23 | *P*= 0.004, B= -1.395 | *r*(2)= 0.291,  *P*= 0.709 |
| *REL* | 288.01 (65.22) | 282.61 (65.50) | 0.98 | *P*= 0.863, B= -6.274 | *r*(2)= 0.686,  *P*= 0.314 |
| *RELA* | 168.35 (21.92) | 174.54 (19.19) | 1.04 | *P*= 0.778, B= -6.244 | *r*(2)= 0.570,  *P*= 0.430 |
| *RELB* | 143.65 (14.66) | 131.40 (24.77) | 0.91 | *P*= 0.459, B= -5.958 | *r*(2)= 0.968,  *P*= 0.0318 |
| *TNF* | 36.42 (8.64) | 34.72 (7.02) | 0.95 | *P*= 0.557, B= -6.079 | *r*(1)= 0.349,  *P*= 0.773 |
| *Type-1 Interferon Response Genes* | | | | | |
| *GBP1* | 463.26 (147.30) | 420.83 (256.33) | 0.91 | *P*= 0.369, B= -5.794 | *r*(2)= 0.820,  *P*= 0.180 |
| *IFI16* | 1135.22 (174.40) | 1199.46 (35.70) | 1.06 | *P*= 0.642, B= -6.160 | *r*(2)= 0.702,  *P*= 0.298 |
| *IFI27* | 60.03 (12.03) | 67.70 (6.84) | 1.13 | *P*= 0.385, B= -5.831 | *r*(2)= 0.124,  *P*= 0.876 |
| *IFI27L1* | 22.77 (1.40) | 28.90 (1.22) | 1.27 | *P*= 0.064, B= -4.229 | *N/A* |
| *IFI27L2* | 139.11 (49.95) | 133.85 (22.35) | 0.96 | *P*= 0.989, B= -6.292 | *r*(2)= -0.414,  *P*= 0.586 |
| *IFI30* | 3630.95 (939.14) | 3947.86 (784.55) | 1.09 | *P*= 0.652, B= -6.166 | *r*(2)= -0.605,  *P*= 0.395 |
| *IFI35* | 164.76 (22.65) | 172.01 (70.66) | 1.04 | *P*= 0.986, B= -6.292 | *r*(2)= -0.370,  *P*= 0.630 |
| *IFI44* | 410.95 (145.43) | 390.52 (259.95) | 0.95 | *P*= 0.475, B= -5.977 | *r*(2)= 0.834,  *P*= 0.166 |
| *IFI44L* | 386.90 (225.78) | 416.09 (457.62) | 1.08 | *P*= 0.524, B= -6.041 | *r*(2)= 0.906,  *P*= 0.094 |
| *IFI6* | 79.78 (32.91) | 92.03 (62.07 | 1.15 | *P*= 0.759, B= -6.234 | *r*(2)= 0.645,  *P*= 0.355 |
| *IFIH1* | 177.62 (33.91) | 165.21 (35.46) | 0.93 | *P*= 0.613, B= -6.136 | *r*(2)= 0.583,  *P*= 0.417 |
| *IFIT1* | 36.93 (2.77) | 63.93 (60.34) | 1.73 | *P*= 0.585, B= -6.108 | *N/A* |
| *IFIT1L* | BDT | BDT | N/A | N/A | N/A |
| *IFIT2* | 103.07 (49.62) | 108.01 (39.70) | 1.04 | *P*= 0.820, B= -6.260 | *r*(2)= -0.0631,  *P*= 0.937 |
| *IFIT3* | 52.91 (7.61) | 88.75 (87.02) | 1.68 | *P*= 0.940, B= -6.289 | N/A |
| *IFIT5* | 100.82 (6.03) | 108.32 (13.79) | 1.07 | *P*= 0.616, B= -6.138 | *r*(2)= 0.415,  *P*= 0.585 |
| *IFITM1* | 928.59 (412.18) | 1321.56 (254.14) | 1.42 | *P*= 0.120, B= -4.809 | *r*(2)= -0.0405, *P*= 0.960 |
| *IFITM2* | 132.23 (28.81) | 127.44 (16.35) | 0.96 | *P*= 0.873, B= -6.277 | *r*(2)= 0.115,  *P*= 0.885 |
| *IFITM3* | 1416.08 (634.74) | 1868.87 (992.82) | 1.32 | *P*= 0.285, B= -5.589 | *r*(2)= 0.353,  *P*= 0.647 |
| *IFITM4P* | 940.06 (357.69) | 1303.47 (410.59) | 1.39 | *P*= 0.120, B= -4.811 | *r*(2)= 0.389,  *P*= 0.611 |
| *IFITM5* | BDT | BDT | N/A | N/A | N/A |
| *IFNB1* | BDT | BDT | N/A | N/A | N/A |
| *IRF2* | 308.92 (85.86) | 318.00 (27.61) | 1.03 | *P*= 0.761, B= -6.235 | *r*(2)= -0.963,  *P*= 0.0374 |
| *IRF7* | 300.35 (69.70) | 253.46 (83.95) | 0.84 | *P*= 0.246, B= -5.473 | *r*(2)= 0.722,  *P*= 0.278 |
| *IRF8* | 655.92 (109.03) | 577.83 (71.88) | 0.88 | *P*= 0.351, B= -5.760 | *r*(2)= 1.00,  *P*< 0.0001 |
| *MX1* | 245.94 (94.59) | 279.82 (177.71) | 1.14 | *P*= 0.712, B= -6.209 | *r*(2)= 0.856,  *P*= 0.144 |
| *MX2* | 505.54 (105.10) | 504.23 (93.58) | 1.00 | *P*= 0.987, B= -6.292 | *r*(2)= 0.317,  *P*= 0.684 |
| *OAS1* | 605.27 (68.24) | 609.02 (116.55) | 1.01 | *P*= 0.980, B= -6.292 | *r*(2)= 0.484,  *P*= 0.516 |
| *OAS2* | 703.57 (28.97) | 770.14 (292.16) | 1.09 | *P*= 0.786, B= -6.247 | *rs*(2)= 0.200, *P*= 0.917 |
| *OAS3* | 180.85 (42.95) | 219.54 (166.82) | 1.21 | *P*= 0.838, B= -6.267 | *r*(2)= 0.718,  *P*= 0.282 |
| *OASL* | 42.52 (4.01) | 59.55 (35.14) | 1.40 | *P*= 0.382, B= -5.821 | *r*(2)= 0.362,  *P*= 0.638 |
| Antibody Synthesis Genes | | | | | |
| *IGJ* | 796.23 (424.95) | 913.01 (607.34) | 1.15 | *P*= 0.628, B= -6.147 | *r*(2)=0.376,  *P*= 0.634 |
| *IGLL1* | BDT | BDT | N/A | N/A | N/A |
| *IGLL3* | BDT | BDT | N/A | N/A | N/A |
| Negative Control | | | | |  |
| *MAPT* | BDT | BDT | N/A | N/A | N/A |
| Note: Baseline to post-treatment differential expression analysis of normalized gene counts carried out with the moderated t-test by Robust Empirical Bayes (REBayes) for paired samples in limma, unadjusted *P* < 0.05. No correction for multiple comparisons was made due to small sample size (N = 2-4). Baseline vs. post-treatment correlations were presented as Pearson r (r) correlations for normally distributed data, and Spearman r (rs) correlations for non-parametric data. N/A indicates too few pairs were available to generate a value. Genes listed by category, then alphabetically; statistically significant *P* values presented in bold. BDT = below detection threshold, < 20, df = degrees of freedom, *FOS* = Fos proto-oncogene, *GBP* = guanylate binding protein gene, *IFI* = interferon-inducible protein gene, *IFIH* = interferon-induced protein with helicase C, *IFIT* = interferon-induced protein with tetratricopeptide repeats, *IFITM* = interferon-induced transmembrane, *IFNB* = interferon-β, *IGJ* = immunoglobulin joining chain, *IGLL* = immunoglobulin λ locus, *IL* = interleukin, *IRF* = Interferon regulatory factor, *JUN* = Jun proto-oncogene, *MAPT* = microtubule-associated protein tau, *MX* = Myxovirus resistance, *NF*κ*B* = Nuclear factor-κB, *OAS* = 2’-5’-oligoadenylate synthetase, OASL = 2’-5’-oligoadenylate synthetase like, *PTGS* = Prostaglandin-endoperoxidase synthase, *REL* = v-rel avian reticuloendotheliosis viral oncogene homolog, SD = standard deviation, *TNF* = tumor necrosis factor | | | | | |
