# Supplementary material for: Evaluation of exploratory fluid biomarkers from a phase 1 senolytic trial in mild Alzheimer’s disease
Source: Neurotherapeutics. 2025 Apr 23;22(4):e00591. doi: 10.1016/j.neurot.2025.e00591 (PMC12418413; doi:10.1016/j.neurot.2025.e00591)
Supplement: Multimedia component 2 [file mmc2.docx]

**Supplementary Figures File.**

**Figure Legends.**

**Supplementary Figure 1: Baseline and post-treatment cerebrospinal fluid Aβ levels measured by mass spectrometry**. **a-d**, Effects of dasatinib plus quercetin (D+Q) on Alzheimer’s disease and related dementia cerebrospinal fluid amyloid β biomarkers (Aβ) measured by mass spectrometry. Baseline to post-treatment changes were assessed using two-sided paired sample t-tests, *P* < 0.05, N = 4, color coded by participant. Mean difference (95% CI): **a**, Aβ42, 0.118 ng/ml (-0.235 to 0.470); **b**, Aβ40 0.70 ng/ml (-3.34 to 4.65); **c**, Aβ42/Aβ40, 0.003% (-0.015 to 0.023). Mean difference = post-treatment – baseline; 95% CI, for the post versus baseline mean difference. **D**, Summary of baseline and post-treatment means, fold change, t-test, P value and 95% CI for each amyloid marker, and the baseline vs. post-treatment correlation. No correction for multiple comparisons was made due to small sample size.

**Supplementary Figure 2. Baseline and post-treatment cerebrospinal fluid tau biomarkers using mass spectrometry. a-j,** Effects of dasatinib plus quercetin (D+Q) on Alzheimer’s disease (AD) and related dementia cerebrospinal fluid tau biomarkers measured by mass spectrometry. Baseline to post-treatment changes were assessed using two-sided paired sample t-tests, *P* < 0.05, N = 5, color coded by participant. Mean difference (95% CI): **a**, pT153/T153%, 0.08% (-0.51 to 0.66); **b**, pT181/T181%, -0.50% (-1.17 to 0.18); **c**, pS199/S199%, -0.03% (-0.13 to 0.07); **d**, pS202/S202%, 0.02% (-0.20 to 0.23); **e**, pT205/T205%, 0.05% (-0.035 to 0.13); **f**, pS208/S208%, -0.01% ( -0.015 to 0.012) **g**, p217/T217%, -0.07% (-1.30 to 1.16); **h**, pT231/T231%, -2.00% (-9.27 to 5.14) **i**, MTBR-T212-221, -0.01 ng/ml (-0.018 to 0.003); **j**, MTBR-T243-254, 0.02 ng/ml (-0.040 to0.073). Baseline to post-treatment changes were assessed using two-sided paired sample t-tests, P<0.05, N = 5, color coded by participant. Mean difference = post-treatment - baseline; 95% CI, for the post versus baseline mean difference. No correction for multiple comparisons was made due to small sample size.

**Supplementary Figure 3. Effects of D+Q treatment on the plasma lipidome**. **a-e**, Effects of dasatinib plus quercetin (D+Q) treatment on the circulating plasma lipidome (related to **Figure 3**). **a**, MetaboAnalyst unsupervised metadata overview (Euclidean distance and Ward clustering) illustrating among all variables tested, biological sex and treatment had the greatest effect on plasma lipidome. Individual subjects follow color coding to match samples across graphs. **b**, Total protein concentrations in plasma and CSF samples assessed by shotgun lipidomics. Paired samples are connected with a line, each color represents a different subject (N = 5). Only *P* < 0.1 are shown. **c**, Interactive PCS plot displaying plasma lipidomic samples color coded by biological sex (females in red, males in green). Note that baseline (circles) and post-treatment (triangles) samples are displayed in different shapes.

**Supplementary Figure 4. Effects of dasatinib plus quercetin (D+Q) treatment on the circulating plasma lipidome normalized to plasma volume.** **a-f**, Effects of dasatinib plus quercetin (D+Q) treatment on the circulating plasma lipidome normalized to plasma volume. Plasma lipidome was assessed using multidimensional mass spectrometry-based shotgun lipidomics. **a**, MetaboAnalyst unsupervised PCA plot reducing all plasma lipid species data into three dimensions. Baseline and post-treatment groups are color-coded in gray and orange respectively, subjects are color coded consistently across figures. **b**, All 11 lipid classes assessed in plasma samples. Paired samples are connected with a line. **c**, Triglyceride (TAG) and **d**, lysophophatidylcholine (LPC) molecular species were grouped into those containing long-chain or very long-chain fatty acyls. **e**, Volcano plot comparing all 194 plasma lipid species at baseline and post-treatment. **f**, Plot of the five differentially abundant lipids (DALs) species significantly altered from baseline to post-treatment. Paired samples are connected with a line, each color represents a different subject (N = 5). Only *P* < 0.1 are shown.

**Supplementary Figure 5. Effects of dasatinib plus quercetin (D+Q) treatment on the cerebrospinal fluid lipidome (CSF), normalized to total protein concentration. a-d,** Effects of dasatinib plus quercetin (D+Q) treatment on the cerebrospinal fluid (CSF) lipidome normalized to total protein content. CSF lipidome was assessed using multidimensional mass spectrometry-based shotgun lipidomics. **a**, MetaboAnalyst unsupervised PCA plot reducing all CSF lipid species data into 3 dimensions. **b**, MetaboAnalyst supervised PLS-DA plot. Baseline and post-treatment groups are color-coded in gray and orange respectively, subjects are color coded consistently across graphs. **c**, Volcano plot comparing all 79 CSF lipid species at baseline and post-treatment. **d**, Plot of the five differentially abundant lipids (DALs) species significantly altered from baseline to post-treatment. Paired samples are connected with a line, each color represents a different subject (N = 5). Only *P* < 0.1 are shown.

| **** | | | | |  |
| --- | --- | --- | --- | --- | --- |
| **Amyloid beta (Aβ) Isoform** | **Baseline**  **Mean (SD)** | **Post-Treatment Mean (SD)** | **Fold Change** | **T-test(df),**  ***P* value,**  **95% CI** | Baseline vs.  Post-treatment Correlation coefficient(df),  *P* value |
| Aβ42 (ng ml^-1^) | 0.853 (0.319) | 0.971  (0.424) | 1.14 | t(3)= 1.058,  *P*= 0.368,  -0.235 to 0.470 | *r*(2)= 0.860,  *P*= 0.140 |
| Aβ40 (ng ml^-1^) | 13.5  (6.07) | 14.2  (7.10) | 1.05 | t(3)= 0.520,  *P*= 0.639,  -3.34 to 4.65 | *r*(2)= 0.939,  *P*= 0.0609 |
| Aβ42/40 (%) | 0.066 (0.010) | 0.069  (0.010) | 1.06 | t(3)= 0.635,  *P*= 0.566,  -0.015 to 0.023 | *rs*(2)= 0.400,  *P*= 0.750 |
| Note: Baseline to post-treatment changes were assessed using two-sided, paired sample t-tests, 95% CI: 95 percent confidence interval for the post versus baseline mean difference, *P* < 0.05. No correction for multiple comparisons was made due to small sample size (N = 4). Baseline vs. post-treatment correlations were presented as Pearson r (r) correlations for normally distributed data, and Spearman r (rs) correlations for non-parametric data. Aβ = amyloid beta; df = degrees of freedom, SD = standard deviation | | | | | |

**Supplementary Figure 1**: Baseline and post-treatment cerebrospinal fluid Aβ levels measured by mass spectrometry.

**Supplementary Figure 2.** Baseline and post-treatment cerebrospinal fluid tau biomarkers using mass spectrometry.


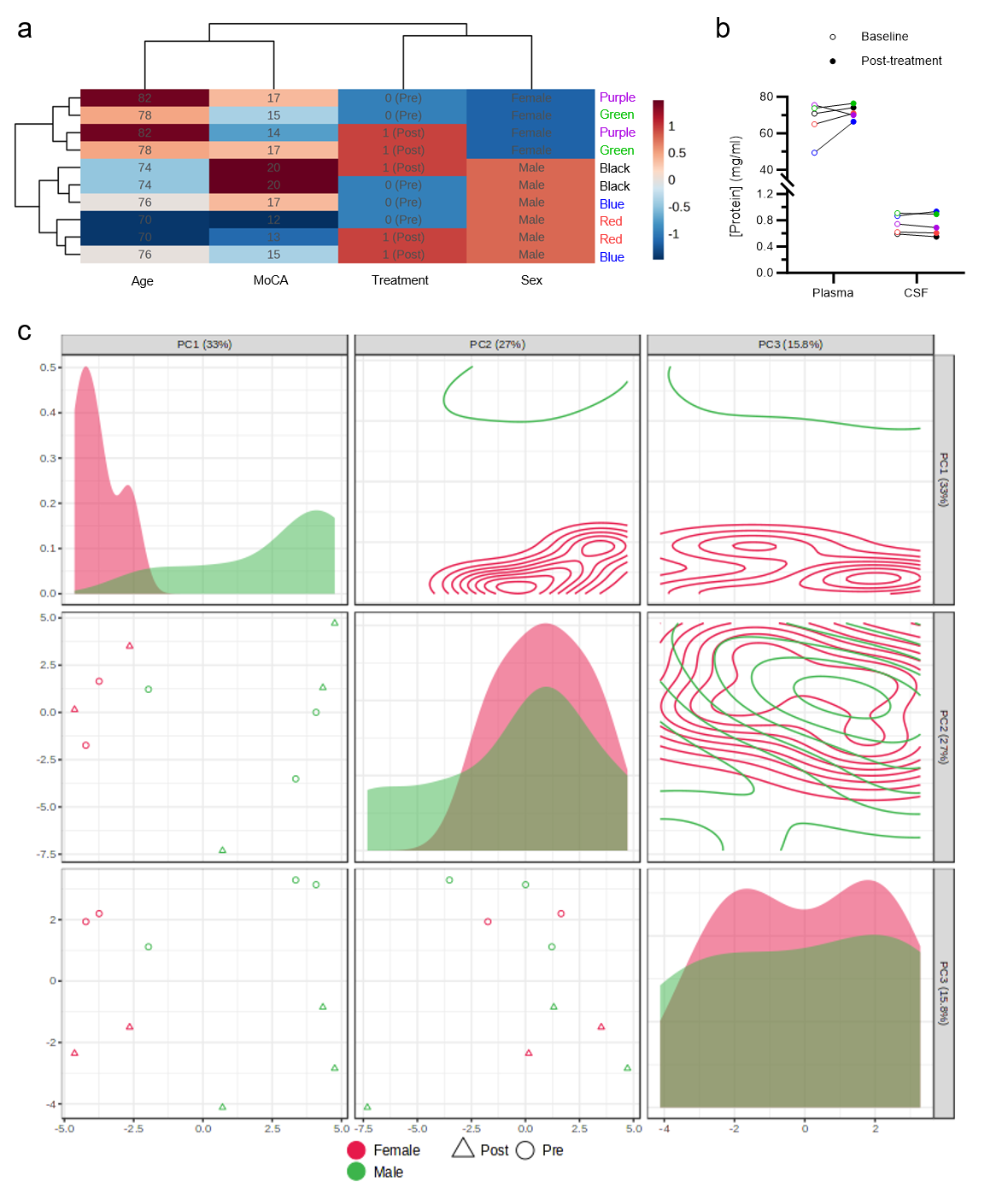


**Supplementary Figure 3**. Effects of D+Q treatment on the plasma lipidome.

**
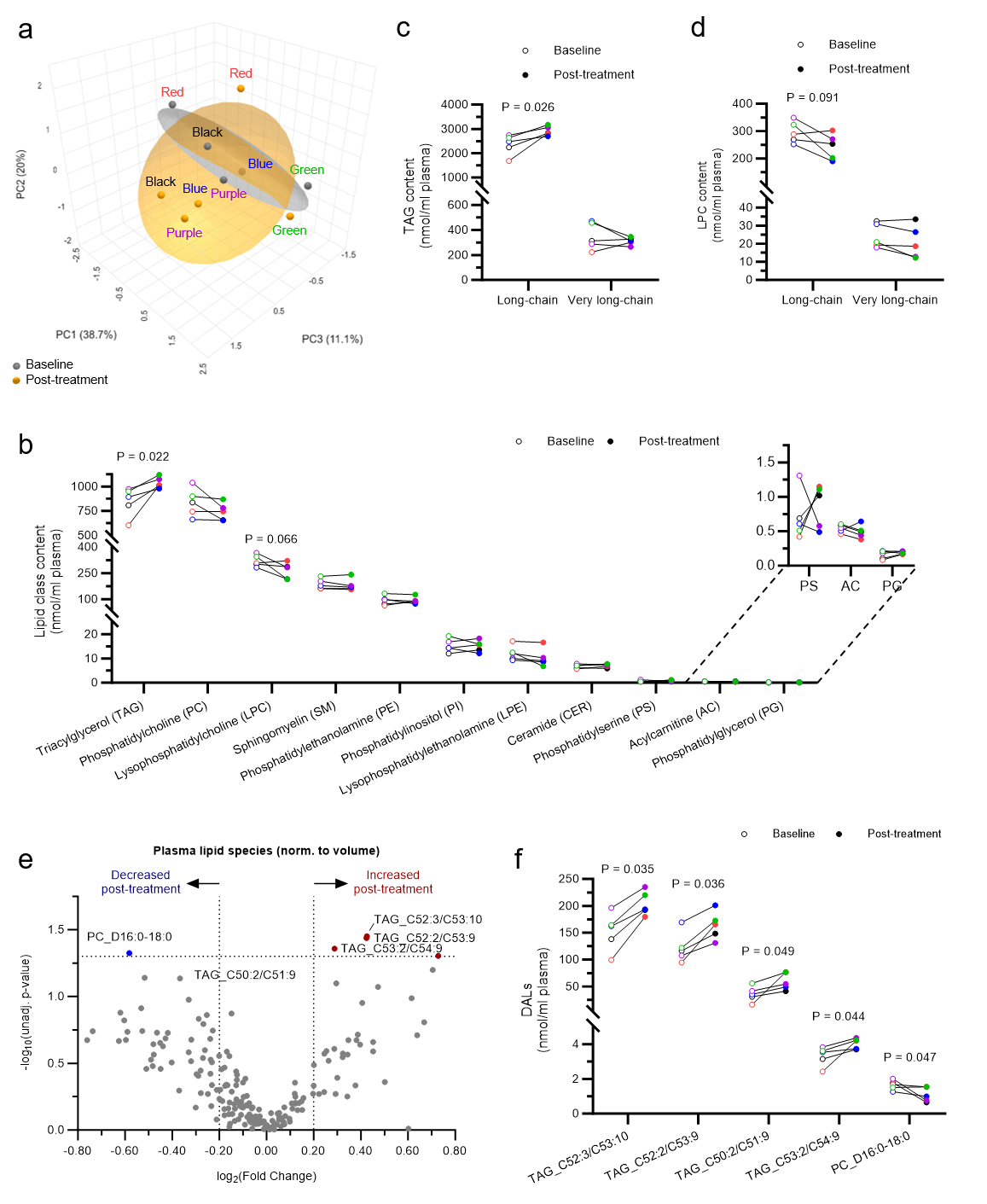
Supplementary Figure 4**. Effects of dasatinib plus quercetin (D+Q) treatment on the circulating plasma lipidome normalized to plasma volume.

**
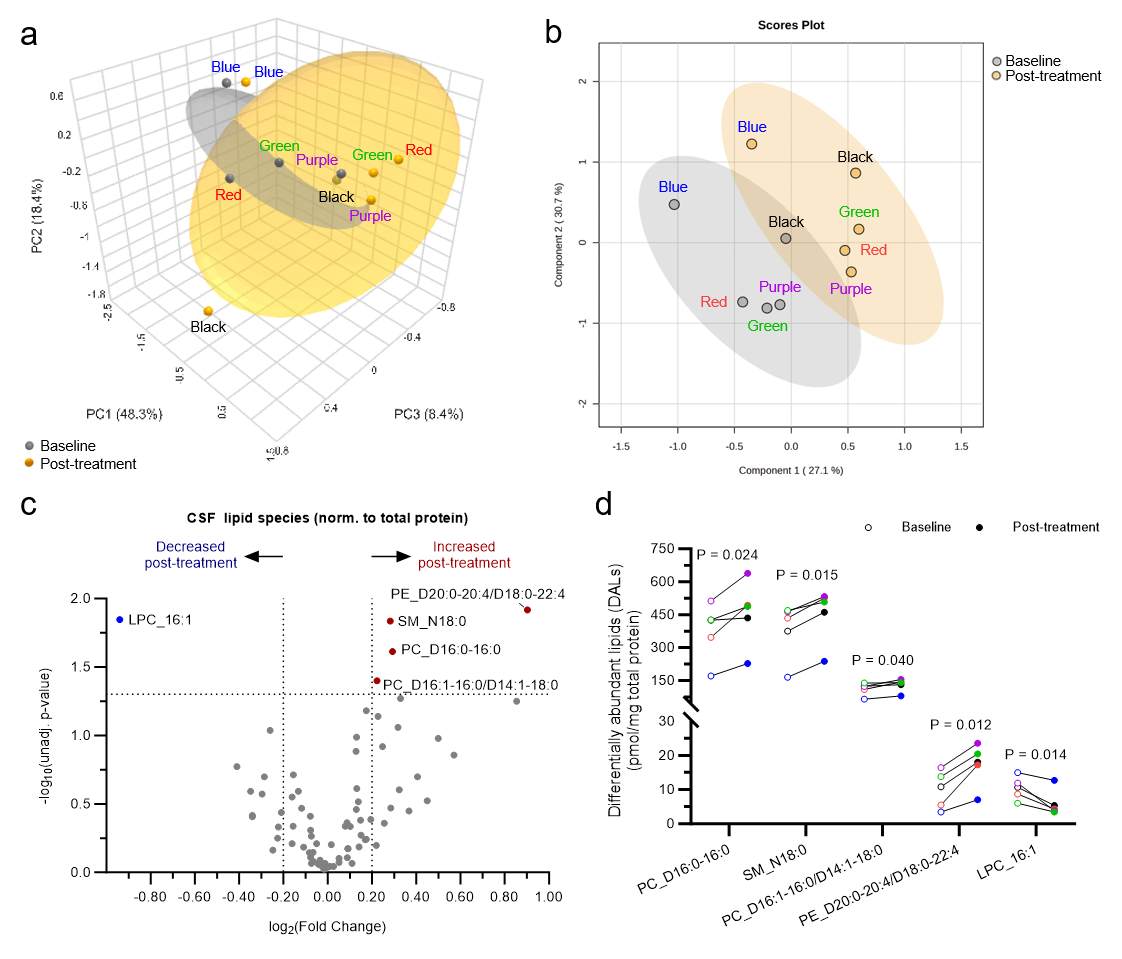
**

**Supplementary Figure 5**. Effects of dasatinib plus quercetin (D+Q) treatment on the cerebrospinal fluid lipidome (CSF), normalized to total protein concentration.
